# Cherenkov luminescence measurements with digital silicon photomultipliers: a feasibility study

**DOI:** 10.1186/s40658-015-0134-z

**Published:** 2015-11-16

**Authors:** Esther Ciarrocchi, Nicola Belcari, Alberto Del Guerra, Simon R. Cherry, Adrienne Lehnert, William C. J. Hunter, Wendy McDougald, Robert S. Miyaoka, Paul E. Kinahan

**Affiliations:** Department of Physics, University of Pisa, Pisa, Italy; INFN, section of Pisa, Pisa, Italy; Department of Biomedical Engineering, University of California, Davis, CA USA; Department of Radiology, University of Washington, Seattle, WA USA

**Keywords:** Cerenkov radiation, Cherenkov radiation, Cherenkov luminescence imaging, Silicon photomultipliers, Digital silicon photomultipliers

## Abstract

**Background:**

A feasibility study was done to assess the capability of digital silicon photomultipliers to measure the Cherenkov luminescence emitted by a *β* source. Cherenkov luminescence imaging (CLI) is possible with a charge coupled device (CCD) based technology, but a stand-alone technique for quantitative activity measurements based on Cherenkov luminescence has not yet been developed. Silicon photomultipliers (SiPMs) are photon counting devices with a fast impulse response and can potentially be used to quantify *β*-emitting radiotracer distributions by CLI.

**Methods:**

In this study, a Philips digital photon counting (PDPC) silicon photomultiplier detector was evaluated for measuring Cherenkov luminescence. The PDPC detector is a matrix of avalanche photodiodes, which were read one at a time in a dark count map (DCM) measurement mode (much like a CCD). This reduces the device active area but allows the information from a single avalanche photodiode to be preserved, which is not possible with analog SiPMs. An algorithm to reject the noisiest photodiodes and to correct the measured count rate for the dark current was developed.

**Results:**

The results show that, in DCM mode and at (10–13) °C, the PDPC has a dynamic response to different levels of Cherenkov luminescence emitted by a *β* source and transmitted through an opaque medium. This suggests the potential for this approach to provide quantitative activity measurements. Interestingly, the potential use of the PDPC in DCM mode for direct imaging of Cherenkov luminescence, as a opposed to a scalar measurement device, was also apparent.

**Conclusions:**

We showed that a PDPC tile in DCM mode is able to detect and image a *β* source through its Cherenkov radiation emission. The detector’s dynamic response to different levels of radiation suggests its potential quantitative capabilities, and the DCM mode allows imaging with a better spatial resolution than the conventional event-triggered mode. Finally, the same acquisition procedure and data processing could be employed also for other low light levels applications, such as bioluminescence.

**Electronic supplementary material:**

The online version of this article (doi:10.1186/s40658-015-0134-z) contains supplementary material, which is available to authorized users.

## Background

Cherenkov luminescence imaging (CLI) [1, 2] is a promising technique to visualize the biodistribution of *β*-emitting radionuclides, and its main advantage is the capability to monitor *β*^−^ emitters, which are used for example in radiotheraphy but are difficult to visualize in other ways, such as Bremsstrahlung imaging [3, 4]. Several studies have shown that an optical system made of a charge coupled device (CCD) with a focusing lens can be used to image the distribution of a *β*-emitting radiopharmaceutical in a mouse (e.g., [5, 6]) or even in the human body through several millimeters of tissue [7, 8]. Other potential applications are Cherenkov luminescence endoscopy and the excitation of fluorophores [9, 10]. Some studies have demonstrated a linear relationship between the Cherenkov signal and the signal measured with positron emission tomography and single photon emission computed tomography (e.g., [11, 12]), thus providing a means to obtain quantitative measurements after this cross-calibration. However, when used as a stand-alone imaging technique, CLI with CCDs has not been able to provide quantitative measurements in terms of number of photons when the Cherenkov radiation is detected in complex media like biological tissue. It has been shown that there are discrepancies between the results of physical experiments and those predicted by Monte Carlo simulations [13, 14], and it has been suggested that these discrepancies are due to the difficulties in modeling factors like the photo-detector quantum efficiency, the transmissivity and aberrations of the optics and the Cherenkov radiator index of refraction. This has been recently confirmed in [15], where for the same reasons only relative comparisons between the measured and simulated data could be obtained.

An alternative to CCDs are silicon photomultipliers (SiPMs), solid state arrays of avalanche photodiodes (APDs) working in Geiger-Muller mode, with single photon counting capability which makes them intrinsically quantitative. SiPMs have been used for different applications [16–20], and are able to detect Cherenkov radiation [21, 22]. In this study we examine the performance of a digital SiPM device, called the Philips digital photon counting device (PDPC) [23, 24], for use in *β*-emission activity measurements by Cherenkov luminescence. The PDPCs are able to directly measure photon count values from the number of triggered cells. The aim of this study was to assess if this kind of photosensor is able to measure the Cherenkov radiation produced by a *β*-emitting source in an optically opaque medium. A ^22^Na point source embedded in a Plexiglas container was used as a Cherenkov radiator, and different materials (chicken breast, black paper, and Plexiglas) were placed between the source and detector to evaluate their effect on the detected signal.

**Fig. 1:**
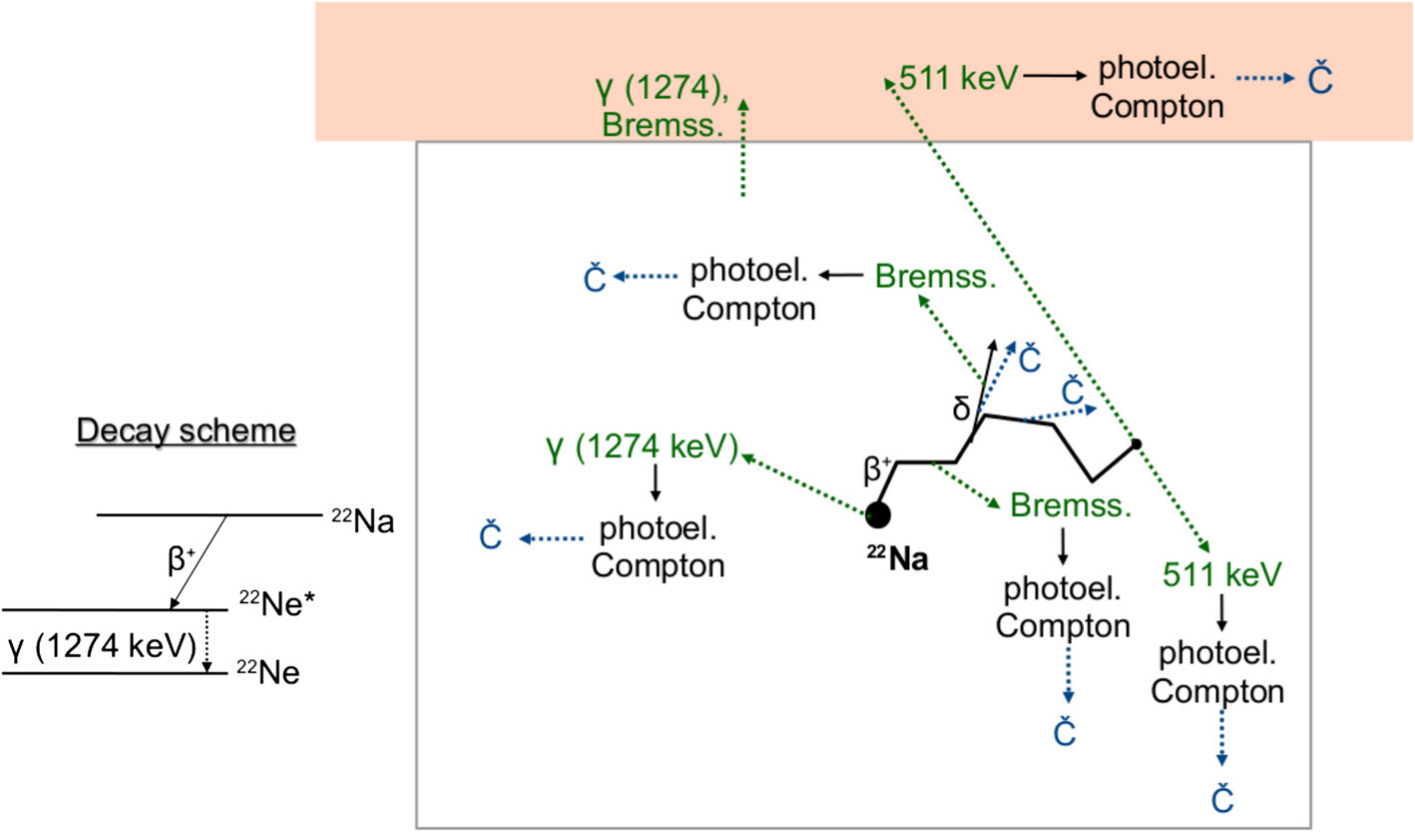
Physics processes resulting in Cherenkov radiation emission. *Left* decay scheme of ^22^Na. *Right* different processes that result in Cherenkov radiation emission: in the source container (*bottom*) and in the external layer of arbitrary thickness (*top*). Abbreviations, *Bremss* Bremsstrahlung, *photoel* photoelectric effect, *δ* = *δ* ray, C̆ = Cherenkov radiation

In a geometry like the one of this study, there are different processes that lead to the production of Cherenkov radiation, as summarized in Fig. 1. The decay scheme of the ^22^Na is shown on the left. The ^22^Na decay produces a positron (with mean energy of 250 keV and maximum energy of 545 keV) and three high energy photons: the two 511 keV annihilation photons and the 1.274 MeV *γ* in the de-excitation of the daughter ^22^Ne nucleus to its ground state. Electron capture is also possible in 10 % of the cases, but it does not produce Cherenkov radiation, and internal conversion as an alternative to the *γ* decay of ^22^Ne has a very low probability (internal conversion coefficients ∼10^−5^−10^−6^, [25]).

In the spectral range of operation of the PDPC (380–700 nm, where the photon detection efficiency is greater than 10 %), Plexiglas has an average refractive index *n* of 1.495 (and a dispersion smaller than 2 %), that corresponds to a Cherenkov threshold of 176 keV for positrons and electrons. Cherenkov radiation can be produced directly by the positrons or by secondary electrons with kinetic energy above this threshold. The secondary electrons can be *δ* rays produced by the positrons in the Plexiglas, or photoelectrons and Compton electrons produced either by the high-energy photons emitted in the source decay or by Bremsstrahlung radiation. Positrons and electrons are stopped by the Plexiglas container, while high-energy photons can travel out of it, so they can interact either in the source volume itself or in the chicken breast (*n*≃1.4), where the Cherenkov threshold is approximately 220 keV. The direct detection of *β* (if any), *γ* and Bremsstrahlung radiation is also possible.

Due to the acquisition mode used for this study, in which the information from the single avalanche photodiode is preserved, an algorithm to subtract the influence of the cells with the highest dark current was developed. The expected dark count rate as a function of the temperature of the photodiode was calculated as well to estimate the true source count rate. As a proof-of-principle, the linearity of the measured count rate with the source activity was tested using ^18^F, and Cherenkov luminescence images of a *β*-emitting source also were acquired.

## Methods

### Data collection

#### Acquisition mode

**Fig. 2:**
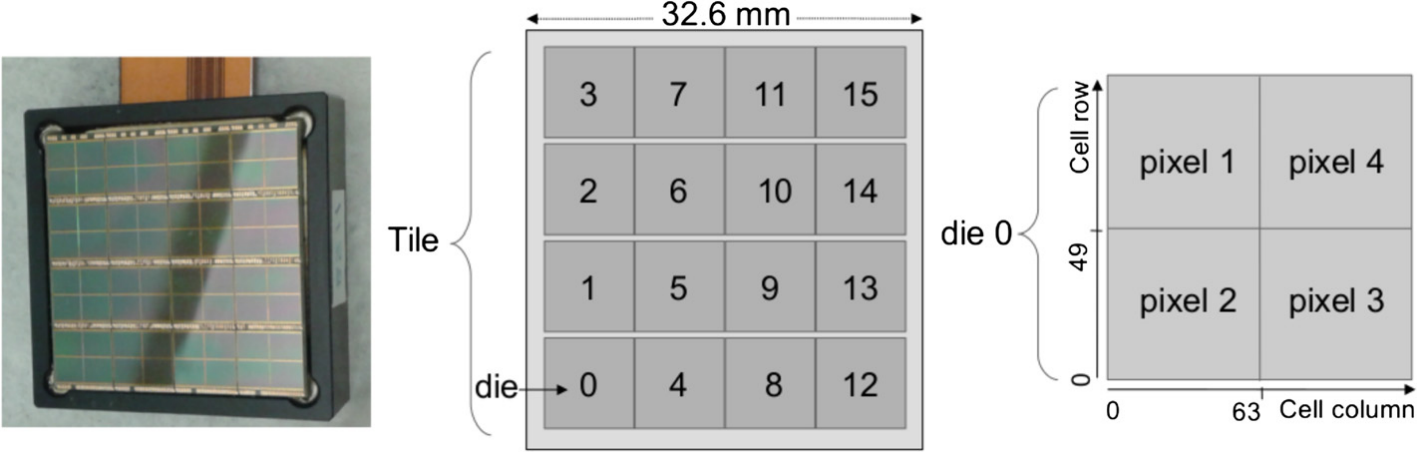
Picture and scheme of the PDPC tile. Scheme adapted from [28]. *Center* The tile is composed of 16 dies arranged in a 4×4 matrix. *Right* Each die is composed of 4 pixels in a 2×2 matrix. Each pixel is made of 64×50 cells, for a total of 204,800 cells in the entire tile

The study was performed using a Philips Digital Photon Counting tile (PDPC, DPC-3200-22 version). A picture of the photo-detector is shown in Fig. 2 (left), together with the scheme of the tile (center) and of a die (right). The tile is composed of 16 dies arranged in a 4×4 matrix. Each die is composed of 4 pixels in a 2×2 matrix. Each pixel is made of 64×50 cells, of 59.4 *μ*m × 64 *μ*m active area, for a total of 204,800 cells in the entire tile. The pixel active area is 3.9×3.2 mm^2^, the pixel pitch is 4.0×4.0 mm^2^, and the tile outer dimensions are 32.6×32.6 mm^2^.

While it is possible to use these devices for Cherenkov radiation measurements in an event-triggered mode [26], in the present work, the PDPCs were used in an event-histogramming mode typically used for calibration to generate the dark count map (DCM). In this mode, one cell per pixel at a time is activated, while the other cells are physically deactivated. The number of photons detected by the cell is summed over a predetermined period called the activation time. Each die is read independently but entirely and row by row every time one of the four simultaneously active cells (one in each of the four pixels in a die) is fired. The output of the acquisition is a DCM, which is a file containing, for every cell of the tile, the length of the activation period, the temperature of the measurement, and the total number of photons counted. The advantage of the DCM mode over the conventional event-triggered mode is that it allows the information on the single cell level to be preserved. When reading cells sequentially, any distortion due to crosstalk is automatically excluded [23]. Usually the DCMs are used to create inhibition maps to mitigate the effects of cells with high dark count rates on noise and dead time. The maps identify the cells with the highest dark current (hereafter called HDC cells), and it is possible to turn off a certain percentage of them during the acquisition.

In this study, the count maps obtained in DCM mode were instead used as raw data. Since in this measurement mode it is not possible to distinguish dark noise photons from photons produced by the source, DCMs were acquired in absence of a source and at different temperatures to calculate the expected dark counts as a function of the cell temperature. Then, the acquisitions with the source were performed, and the calculated dark noise value was subtracted to correct for the background, as described next.

Each cell was activated for either 65 or 98 ms during the DCM acquisition, and for 98 ms during the SCM acquisition. Count values were rescaled to count rates according to the different activation time. Since each pixel contains 64×50 cells, and one cell per pixel was activated at a time, the total acquisition time to scan the entire tile in DCM mode was 3.5 or 5.2 min.

#### Arrangements for activity measurements

**Fig. 3:**
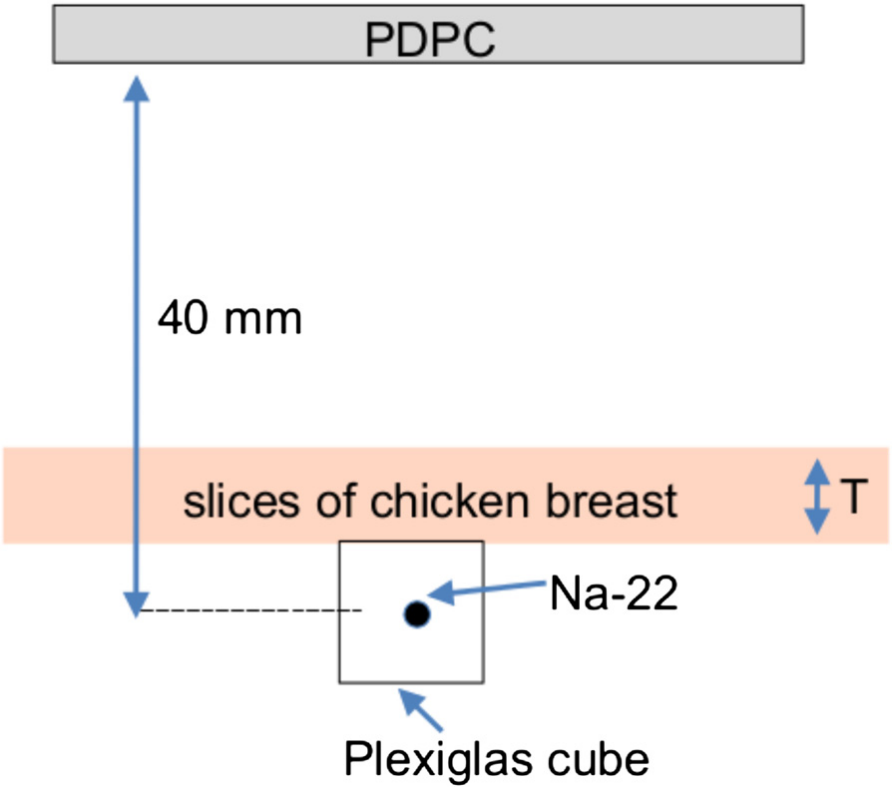
Setup used in this study. A ^22^Na point source embedded in a transparent plastic cube was used as a Cherenkov radiator, and tissue slices of different thickness (*T* = 2–9 mm) were used as an attenuator

All measurements were acquired in a light-tight chamber, and the PDPC tile was air cooled to (10–13) °C. The temperature variations in this range are due both to fluctuations of the air cooling system and to the increase of temperature of the tile during the acquisition shown in [27]. A 25.5 MBq (690 *μ*Ci) ^22^Na point source embedded in a clear Plexiglas cube of 1 cm side was used as the Cherenkov radiator; this choice was convenient for a first feasibility study since clear Plexiglas allows both the production (it has a refractive index *n*≃1.495) and the transmission of the Cherenkov radiation (90 % transmission in the visible range). The distance between the source and the entrance window of the photosensor was approximately 40 mm. Following the approach of [6], chicken breast tissue slices of different thickness were placed on top of the source as illustrated in Fig. 3 to assess the effect of the increasing attenuation of the tissue. To evaluate the importance of some of the contributions illustrated in Fig. 1, acquisitions with different arrangements were performed, as shown in Fig. 4:

1. an acquisition with the source alone was used as a reference;

**Fig. 4:**
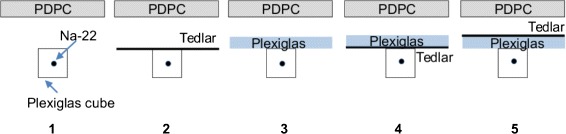
Arrangements used to separate components of the signal
2. a thin black Tedlar sheet (a polyvinyl fluoride film with strong UV attenuation) was used to suppress the Cherenkov radiation contribution only;
3. a piece of Plexiglas of about 3 mm thickness was used to stop *β* particles leaving the source, without affecting too much the Cherenkov signal (it should be noted that the 5 mm of Plexiglas in the source container should be already sufficient to stop the *β* radiation of ^22^Na);
4. the combination Tedlar + Plexiglas was used to stop the Cherenkov radiation produced in the source but not that produced in the outer Plexiglas layer from the positrons/electrons (if any) or high-energy photons that reached it;
5. the combination Plexiglas + Tedlar was used to stop both contributions.

The Tedlar and Plexiglas layers were used in alternate orders (arrangements 4 and 5) to evaluate whether or not there was Cherenkov radiation produced out of the source volume. If such was the case, in case 5, the amplitude of the signal would be smaller than in 4. The remaining component in case 5 would be an indication of the importance of the direct detection of high energy photons.

#### Test of count rate linearity with activity

To test the linearity of the measured count rate with respect to activity, 100 MBq (2.74 mCi) of ^18^F-FDG was diluted in about 2 ml of water and placed in a Petri dish, about 5 mm away from the PDPC tile. Source count maps were acquired over a period of approximately 3 half lives (*t*_1/2_=110 min for ^18^F).

#### Arrangement for image acquisition

**Fig. 5:**
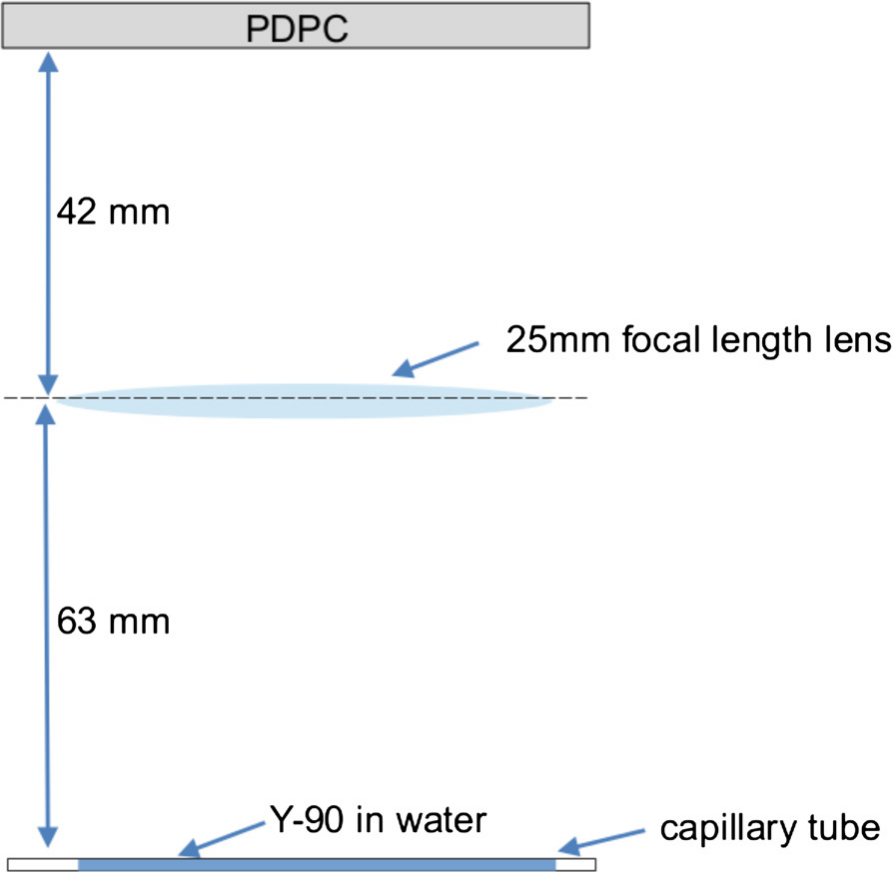
Arrangement used for the image acquisition (figure not to scale)

A focusing lens with 25 mm focal length was used with the PDPC detector in DCM measurement mode to image a capillary tube, filled with 11.1 MBq (300 *μ*Ci) of ^90^Y (a pure *β*^−^ emitter) diluted in water, with the arrangement illustrated in Fig. 5 (figure not to scale). The capillary was 75 mm long, with an active length of 36 mm and an outer radius of 1.7 mm. The distance from the lens was 63 mm, while that between the lens and the entrance window of the photosensor was 42 mm. Slices of chicken breast of 1 and 3 mm thickness were placed on the capillary tube to serve both as Cherenkov radiator and attenuator. In this case, new DCMs were acquired to account for the focusing effect of the lens on the background light possibly leaking into the dark chamber.

### Data analysis

**Fig. 6:**
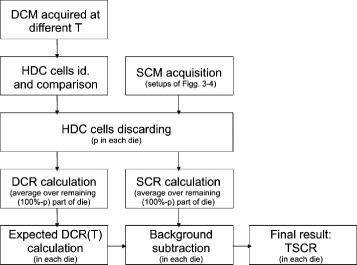
Data processing flow. Procedure for dark count maps (*left*), and for source count maps (*right*). Acronyms, *DCM* dark count map, *SCM* source count map, *p* percentage of inhibition (fraction of HDC cells discarded), *DCR* dark count rate, *SCR* source count rate, *T* average die temperature

The different steps of the data processing are summarized in Fig. 6.

#### Dark count maps

To improve the signal-to-noise ratio, we used the approach of excluding the percentage *p* of the most active cells, also called the inhibition level. First, for a chosen percentage of inhibition *p*, the HDC cells in each die were determined for each dark count acquisition. To understand if the HDC cells identification was reliable, the labels of the selected cells were compared to determine how many of them were different between the measurements. The number given by this comparison was then divided by the total number of HDC cells to obtain the percentage of variation of the HDC cells as a function of the percentage of inhibition *p*. Then the effect in terms of noise reduction of excluding the percentage *p* of the most active cells was investigated by determining the cell dark count rate as a function of the percentage *p*, in this case by averaging over the entire tile.

After that, the dark count rates (DCR) measured with 65 or 98 ms activation times were compared. The DCMs with *t* = 98 ms were used to calculate the expected DCR as a function of temperature and inhibition level as described below (Fig. 6, left), and the DCMs with *t* = 65 ms were used as a test (Fig. 6, right). Then the DCMs collected with both activation times were used to form two new sets of DCM acquisitions, independent of activation time, to test the algorithm of Fig. 6. This was done since the 98 ms DCMs were acquired over a limited temperature range compared to the 65 ms DCMs. Again, the first set was used to derive the parameters for predicting the DCR (Fig. 6, left) as described next, and the second DCM set was used as a test (Fig. 6, right).

A dark count map of the first set, acquired at the average temperature of the entire set of acquisitions, was used to identify the percentage *p* of HDC cells and to exclude them from every other dark count maps. From the first dark data set, the expected dark count rate as a function of temperature was obtained for each die, averaging over the remaining fraction (100 *%*−*p*) of cells of that die, with a linear fit:

(1) $$\begin{array}{@{}rcl@{}} {DCR}_{\text{die,p}}(T) = a <T>_{\text{die,p}} + \,b \end{array} $$

where *DCR*_die,p_ is the average number of dark noise photons per second detected by the cells in the die at inhibition *p*, and <*T*>_die,p_ is the average temperature of the die during the measurement at inhibition *p*. Finally, all DCMs were merged together to correct the SCM with this method, explained in Fig. 6.

#### Source count maps

The same dark count map chosen previously was used to select the HDC cells to discard in each SCM. The source count rate *SCR* was calculated for each die, averaging over the values measured by the (1−*p*) remaining fraction of cells, and the calculated *DCR* value was subtracted from it in each die, to estimate the true source count rate (*TSCR*):

(2) $$\begin{array}{@{}rcl@{}} {TSCR}_{\text{die,p}}(T) = {SCR}_{\text{die,p}}(T) - {DCR}_{\text{die,p}}(T) \quad \quad \forall \; \text{die} \end{array} $$

where *T*= <*T*>_die,p_ is the average temperature of the die during the acquisition of the SCM, at inhibition *p*.

#### Test of count rate linearity with activity

The ^18^F-FDG SCMs were corrected with the algorithm of Fig. 6, and the measured *TSCR* of Eq. 2 was plotted, for each die and for *p*=25 *%*, as a function of the source activity at the time *t* of the acquisition, calculated as:

(3) $$ A(t) = A_{0} \cdot \mathrm{e}^{-\frac{t}{\tau}}  $$

with *A*_0_=2.74 mCi and *τ*=*t*_1/2_/ln(2). A linear fit with parameters *m* and *q* was used to test linearity in each die:

(4) $$ {TSCR}_{\text{die},25\,\%} (t) = m_{\text{die},25\,\%} \cdot A(t) + q_{\text{die},25\,\%}.  $$

#### Images

New DCMs were acquired to account for the focusing effect of the lens on any background light leaking into the dark chamber, and the overall impact of the lens was evaluated by estimating the percent increase in the dark count rate (DCR). The DCMs and the SCMs acquired with the capillary tube source were arranged in images of 512 × 400 cells each. Subsequent analysis was performed on the images rather than on each die. The expected dark count rate as a function of temperature of Eq. 1 was calculated in every cell rather than as an average on the die, and the background subtraction of Eq. 2 was performed cell by cell. Since no inhibition was applied (*p* = 0 %), HDC cell exclusion was accomplished by using 3×3 cells median filters applied to the final images.

## Results

### High dark current (HDC) cells variation

The variation of which cells exhibited the highest dark current as a function of the percentage of inhibition *p* is shown in Table 1. When turning off no more than *p*=25 *%* of the die, the fraction of HDC cells that changed through the measurements was not greater than 4 %, while it increased for higher values of *p*, e.g., up to 20 *%* for *p*=50 *%*.

**Table 1:** HDC cells variation. Percentage of HDC cells that varied between dark count acquisitions as a function of the chosen inhibition percentage *p*, across the limited temperature range (10–13 °C) examined in this study

*p* (%)	5	10	20	25	30	35	50
% of varying HDC cells	2	3	4	4	6	15	20

### Dark count rate vs. inhibition threshold

**Fig. 7:**
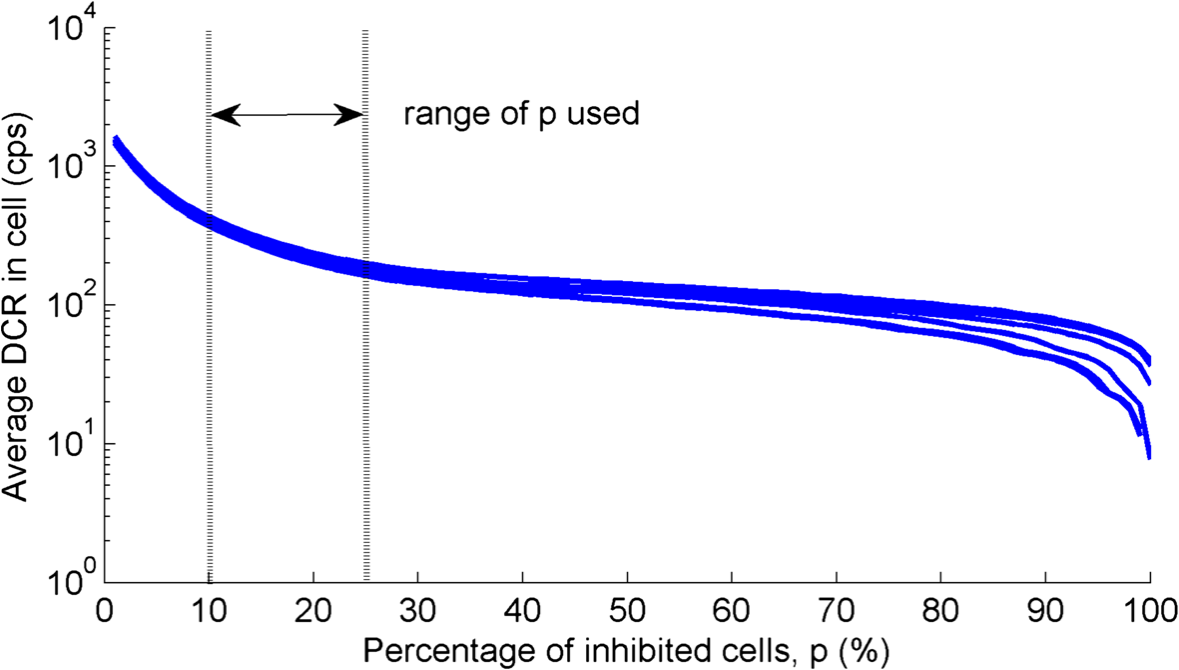
Dark count rate dependence on tile inhibited area. Average dark count rate in a cell of the PDPC as function of the percentage of inhibition *p*, for all the DCMs acquired at different temperature in the range (10–13) °C (*different curves*)

The reduction in the average dark count rate in a cell is shown in Fig. 7 as a function of the inhibition threshold *p* for all the DCMs acquired. The differences between the curves are due to the different temperature of the acquisition (in the range 10–13 °C), and they are relevant only at high inhibition thresholds *p*>25 *%*. By using the information in the entire tile (*p*=0 *%*), the average dark count rate in a cell is of about 1550 cps. This average dark count rate drops down to approximately 400 and 180 cps by discarding, respectively, *p*=10 and 25 % of the most active cells. The optimal percentage of inhibition depends on the expected source count rate, which is not known in our setup. However, for *p*<10 *%* the dark count rate is still high, while for *p*>25 *%*, there is a loss in active area but not a significant reduction of the DCR (less than 30 % reduction going form *p*=25 *%* to *p*=50 *%*). For these reasons, we decided to limit our investigations to values of 10 *%*<*p*<25 *%* (indicated area in Fig. 7).

### Count rate distribution for dark and source data sets

**Fig. 8:**
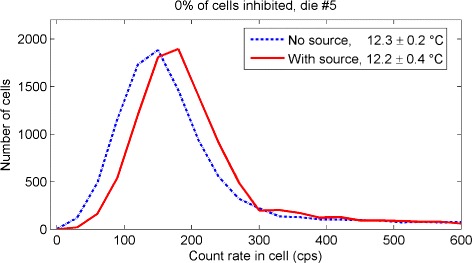
Comparison of the count rate with and without source. Distribution of the count rate measured by the cells of one die at the same temperature for an acquisition with no source (*dotted line*, *T*=12.3±0.2 °C) and for an acquisition with the source (*solid line*, *T*=12.2±0.4 °C). All the cells were considered (no HDC cells inhibition). The maximum measured count rate was about 10^5^ cps. However, the plot was truncated to show details at lower count rates

Figure 8 shows the distribution of the count rate measured by the cells of a die for one data set with no source (dotted line) and for one source data set (solid line), with no inhibition *p* applied. The two acquisitions were performed at similar temperatures (*T*=12.3 ± 0.2 °C and *T*=12.2 ± 0.4 °C, respectively); therefore, a qualitative comparison is possible. Since HDC cells extend the tail to values up to 10^5^ counts per second (cps), the plot is cut at 600 cps. The effect of the source is to shift the distribution to higher values of count rate. In particular, the value of count rate corresponding to the mode of the distribution is increased by 20 %, from 150 to 180 cps. Results for all dies are shown in Additional file 1.

### Variance in measured count rates

**Fig. 9:**
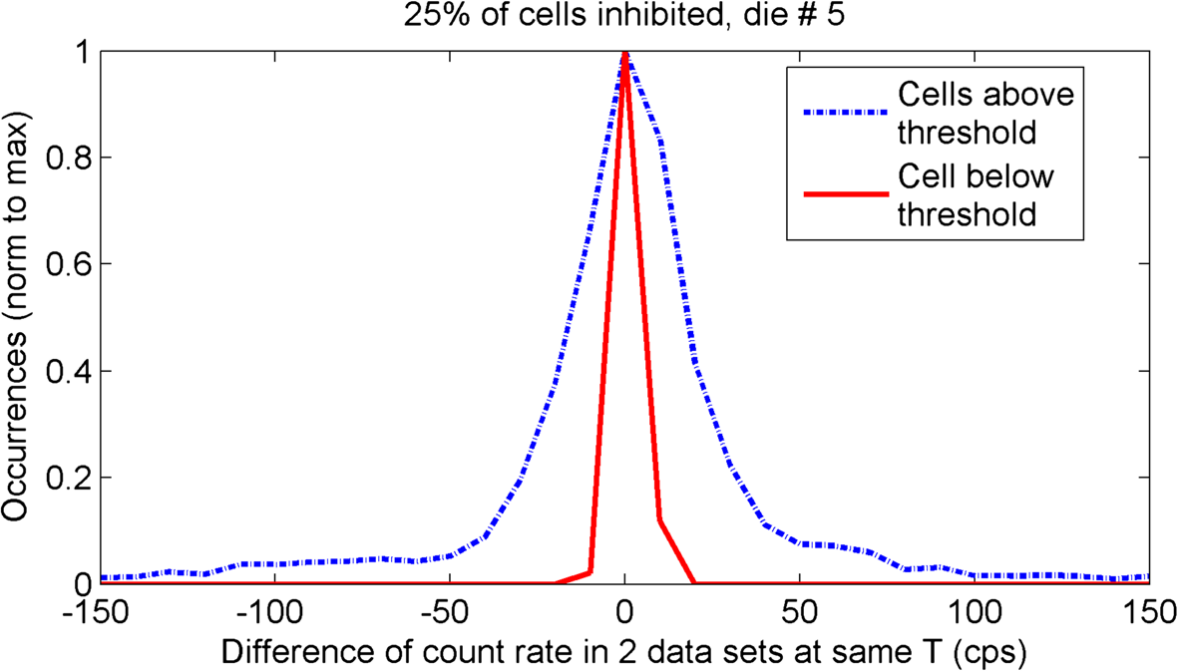
Comparison of measurements at same temperature. Distribution of the differences in the count rates measured in two independent acquisitions performed at the same temperature (12.5±0.4 °C), for one central die of the tile

Two test-retest dark acquisitions were performed at the same temperature to evaluate the variance of the measured count rates. Figure 9 shows the distribution of the difference between the count rates measured by each cell of one die in the two acquisitions (results for all dies are shown in Additional file 2). The dotted line shows the distribution for the 25 *%* HDC cells of the die, while the continuous line is for the remaining 75 *%* cells. Since this second set of cells was used for the count rate calculation, the standard deviation of this ensemble was used as an estimate of the error on the measured count rates. A different value of the standard deviation was calculated for each die and for each percentage of inhibition, and the results are summarized in Table 2. The standard deviation of the remaining (100 *%*−*p*) cells is reduced on average over all dies by 50 % by increasing *p* from 10 to 25 % since this process removes a greater portion of the tail of the distribution of count rates (see Fig. 8), with a consequent narrowing of the fluctuations of the measured count rate (Fig. 9).

**Table 2:** Standard deviation in measured count rates. Standard deviation of the distribution of count rates (cps), for each die and for different values of *p*

	*σ*_*CR*_(cps)					
	*p (%)*					
*Die#*	0	5	10	15	20	25
0	43	11	6	5	4	3
1	44	11	5	5	4	4
2	44	14	6	5	4	3
3	44	13	7	5	4	3
4	45	11	6	5	4	4
5	45	12	7	5	4	3
6	38	14	6	4	4	3
7	43	13	7	5	4	4
8	38	11	6	4	3	3
9	41	13	6	5	3	3
10	43	10	6	5	4	4
11	43	11	6	5	4	3
12	46	12	5	4	3	3
13	45	11	6	4	3	3
14	43	12	6	4	4	3
15	42	13	8	6	4	3
mean ± std	43 ±2	12 ±1	6 ±1	4.7 ±0.6	3.7 ±0.4	3.2 ±0.4

### Comparison of DCMs with different activation time

**Fig. 10:**
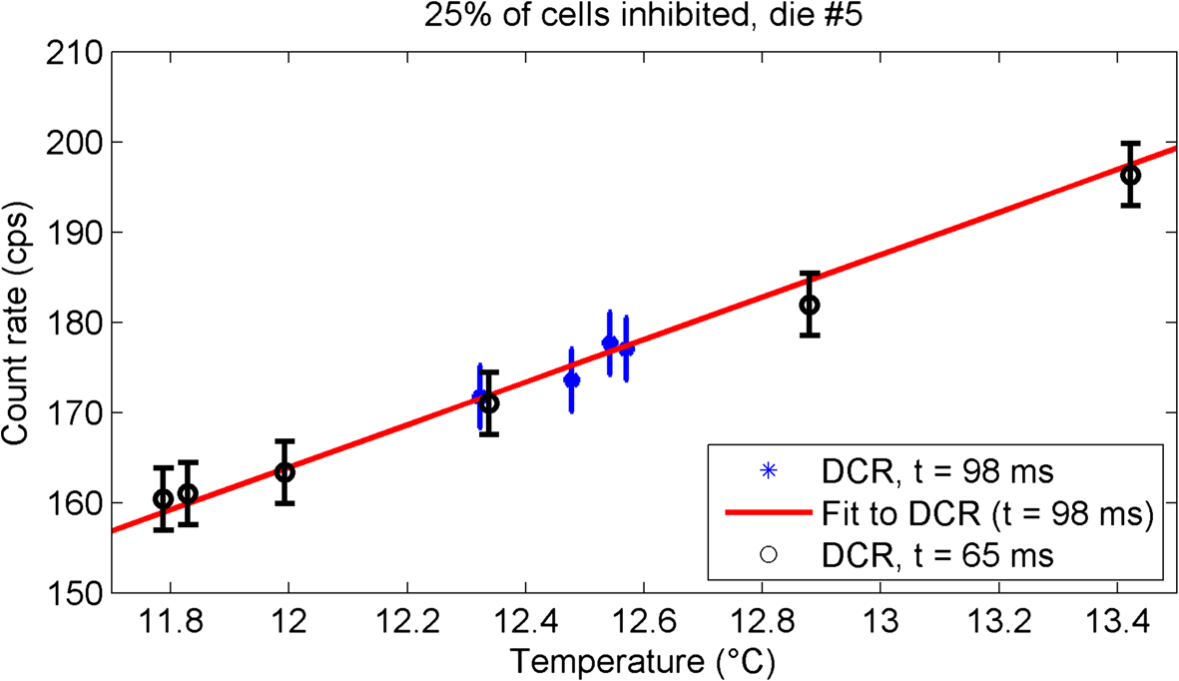
Comparison of the dark count rate with different activation time. Dark count rate measured with *t*=98 ms (*stars*), expected dark count rate as a function of temperature (*solid line*), data set with *t*=65 ms (*circles*)

The comparison of the DCMs acquired with the two activation times (65 and 98 ms) is shown in Fig. 10 for one central die. Results for all dies are shown in Additional file 3. The data set with *t*=98 ms (stars) was used to calculate the expected dark count rate as a function of temperature (Eq. 1, solid line), which predicts properly the trend of the data set with *t*=65 ms (circles). Since there was no apparent impact from the choice of activation time, both sets (65 and 98 ms) were merged together to provide improved sampling over a broader temperature range. The fit to the combined data, Eq. 1 was used to correct the SCM acquisitions, which used a 98 ms activation time.

### Test of HDC cells exclusion algorithm

**Fig. 11:**
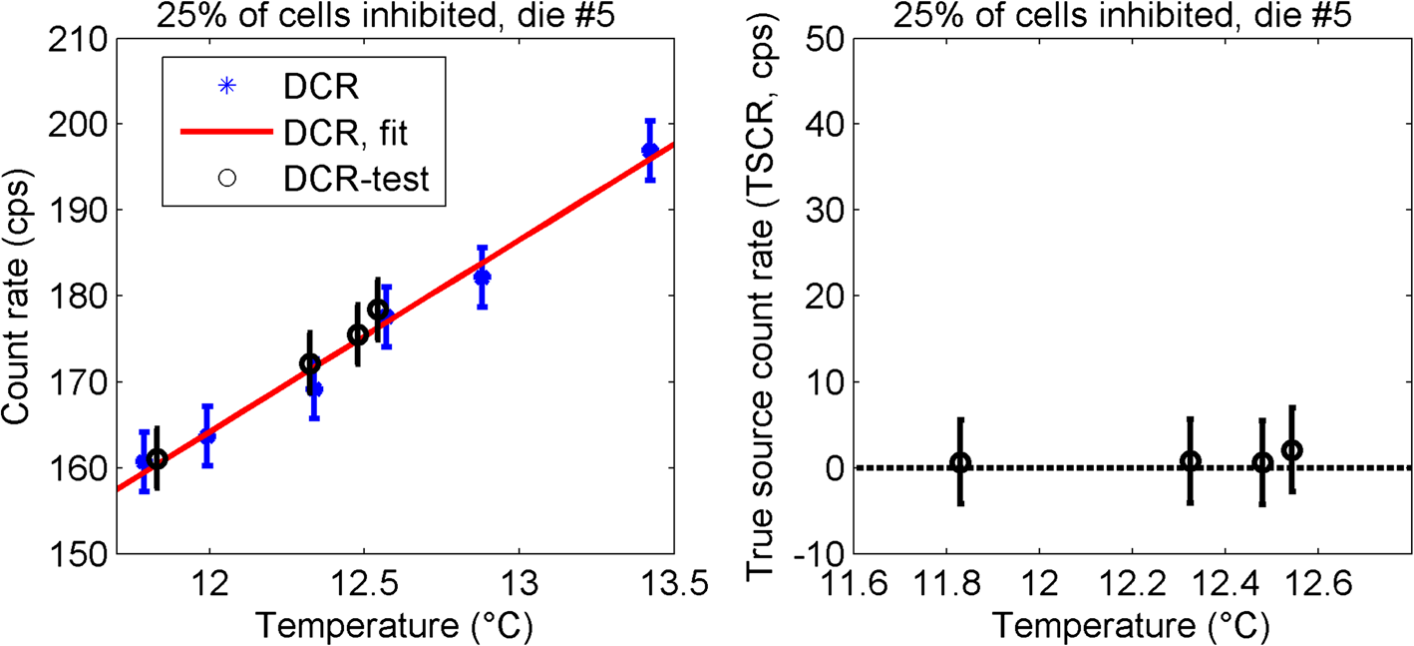
Test of correction algorithm. Example of application of the correction algorithm to the test set without the source. *Error bars* are taken from Table 2

The dark count map acquired at a temperature of approximately 12.5 °C was chosen to identify the HDC cells and to exclude them in all the remaining maps (without and with the source, DCMs and SCMs). The algorithm of Fig. 6, right was first tested on the test set without the source, to prove that the background correction was reliable. The result is shown in Fig. 11 for *p*=25 *%*, and for one central die, the measured dark count rate as a function of temperature is shown on the left for the dark data set (stars), together with the linear fit (solid line), and for the test set without the source (circles). The result of the background subtraction for the test set without the source is shown on the right. After removal of the HDC cells and the background subtraction, the resulting count rate is zero. The same results were obtained for every die (as shown in the Additional files 4 and 5).

### HDC cells exclusion and dark count rate correction

**Fig. 12:**
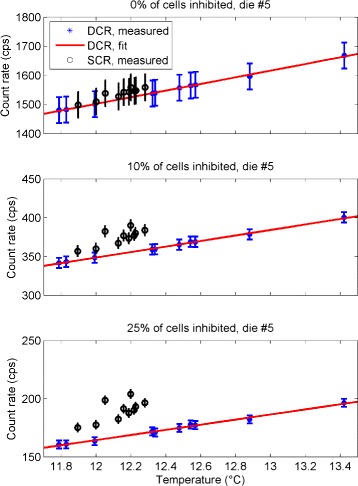
Effect of the HDC cells removal algorithm. *Error bars* are taken from Table 2. The *y*-scale is different for each plot, and the dispersion on the *y*-axis of the data related to the source is due to the different attenuation of the light during the acquisition (different thickness of chicken breast, see Fig. 3)

The effect of the dark count rate correction algorithm on the SCM data is shown in Fig. 12 for one of the central dies of the tile. The top plot shows the uncorrected results for the dark count rate (DCR) and the source count rate (SCR) as a function of temperature. The solid line is the result of the fit operation described by Eq. 1. The central and bottom plots show the same data after the inhibition of the 10 and 25 % HDC cells in each die. The dispersion of the SCR on the *y*-axis is due to different conditions in which the acquisition was performed: each point corresponds to a measurement with an attenuator of different thickness, as shown in Fig. 3; therefore, the amount of light transmitted is different. The *y* scale is different for each plot since the contribution of the HDC cells strongly influences the average count rate in the die. Results for all dies are shown in Additional files 6, 7, and 8.

The estimated coefficients for Eq. 1 are shown in Table 3 as a function of the percentage of inhibition of the HDC cells, after averaging over the 16 dies of the tile. The linear fit follows the trend of the experimental data in the small range of temperatures involved. However, it does not describe the temperature dependence of the DCR on a broader range; for example, at *T*=0 °C, it predicts a negative DCR.

**Table 3:** Dark count rate fit coefficients. Estimated coefficients for the dark count rate as a function of temperature for different percentages of inhibition. The values shown were fitted for each die and then averaged over the 16 dies

*p* (%)	a (cps/ °C)	b (cps)
0	106 ± 6	97 ± 22
10	35 ± 2	−71 ± 10
25	21.6 ± 0.9	−96 ± 8

### Effect of attenuator

**Fig. 13:**
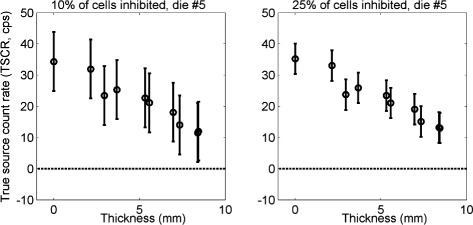
Effect of attenuator. Effect of the attenuation of the chicken breast placed between source and detector, with the setup shown in Fig. 3 in one central die, for *p*=10 *%* (*left*), and *p*=25 *%* (*right*)

Figure 13 shows the estimated true source count rate of Eq. 2 as a function of the thickness of chicken breast positioned between the source and the detector, with the setup shown in Fig. 3. The plot shows the results for a central die, after the HDC cells exclusion (with *p*=10 *%* and 25 *%*) and the dark count rate subtraction. The first measurement in the plots is the reference with the source alone, that produces about 35 cps. The addition of an attenuator rapidly reduces the count rate, down to about 13 cps for 8 mm thickness (roughly a 40 % reduction). The error on the calculated value is reduced by 50 % by increasing *p* from 10 to 25 % after the propagation of the error of Table 2. Results for all dies are shown in Additional files 9 and 10.

### Effect of materials

**Fig. 14:**
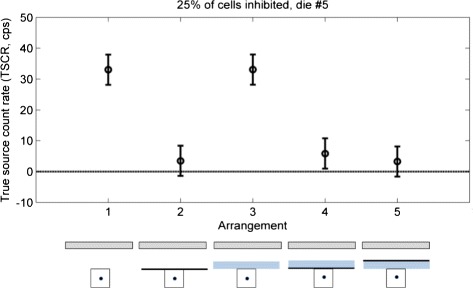
Effect of materials. Labels follow those of Fig. 4
- the addition of Plexiglas (Arrangements 3, 4, 5) does not cause a measurable change in counts;
- the order of Tedlar and Plexiglas (Arrangements 4 and 5) does not make a measurable difference.

Figure 14 shows the different contributions to the final measured count rate. The different cases were labeled according to Fig. 4, and they show that:

- the use of the Tedlar sheet alone or in combination (Arrangements 2, 4, and 5) causes a strong suppression of the global signal with respect to the source alone (Arrangement 1);

Results for all dies are shown in Additional file 11.

### Count rate linearity with activity

**Fig. 15:**
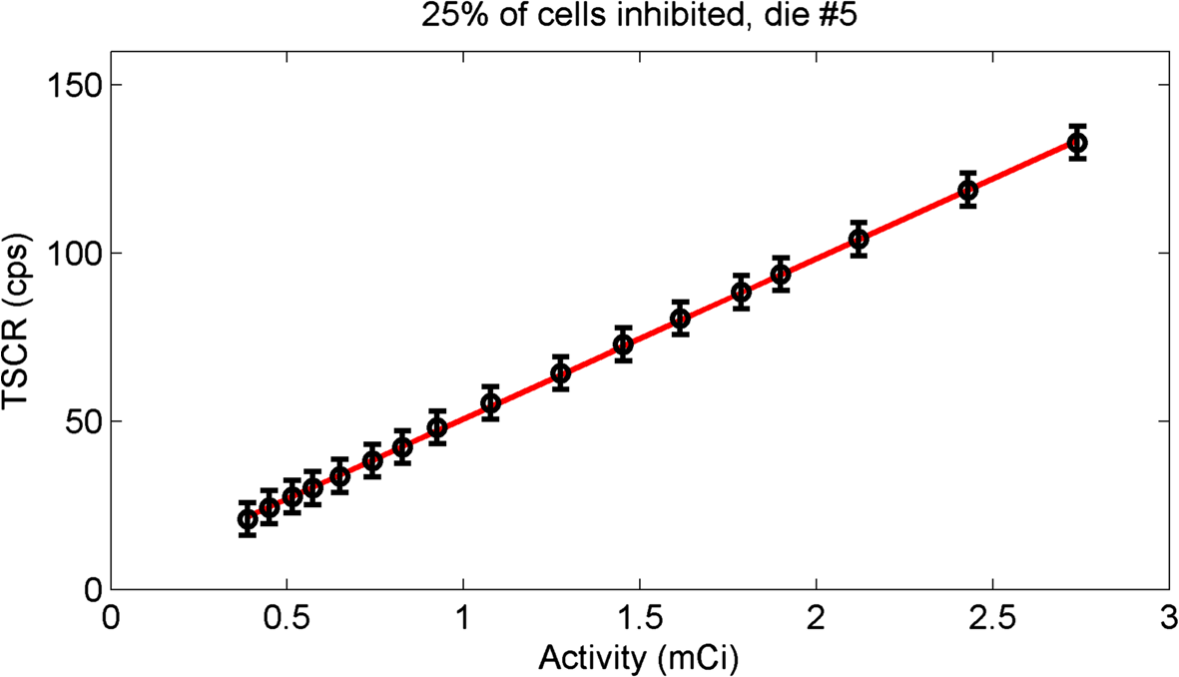
Linearity of count rate with activity. TSCR measured in one central die as a function of the source activity at the time of the measurements (*circle*). Linear fit of Eq. 4 (*solid line*)

The results of the linearity evaluation are shown in Table 4 for *p*=25 *%*, and the TSCR measured in time in one central die as a function of the activity calculated with Eq. 3 is shown in Fig. 15 (circles) together with the result of the linear fit of Eq. 4 (solid line). Results demonstrate a linear response to different levels of activity. Results for all dies are shown in the Additional file 12.

**Table 4:** Count rate linearity with activity. Estimated coefficients for the true source count rate as a function of the ^18^F activity in time for *p*=25 *%*

Die#	m (cps/mCi)	q (cps)
0	31	1.7
1	41	2.5
2	42	2.8
3	33	2.2
4	40	2.4
5	48	3.2
6	48	3.7
7	41	3.3
8	38	1.9
9	46	3.6
10	47	3.5
11	41	2.8
12	25	0.6
13	37	2.8
14	39	2.1
15	26	1.8
mean ± std	39 ±7	2.6 ±0.8

### Images

The DCR calculated using Eq. 1 with the lens added to the system was compared to that measured without the lens for *p*=25 *%*, and average results over the temperature range of the image acquisitions (11.7–13.5) °C are shown in Table 5. The percentage of inhibition *p*=25 *%* was used to highlight the differences between the two data sets. The average 4 % increase of the DCR is presumably due to light leaking into the dark chamber and focused by the optics.

**Table 5:** Percent increase in DCR due to focusing lens. DCR_*nl*_ and DCR_*l*_ are the expected DCR without and with lens, for *p*=25 *%*. Values are averaged over the temperature range (11.7–13.5) °C of the image acquisitions

	Die#	$\frac {\text {DCR}_{l} - \text {DCR}_{\textit {nl}}}{\text {DCR}_{\textit {nl}}}$ (%)	
	0	5.2±0.4	
	1	3.45±0.01	
	2	2.9±0.3	
	3	4.8±0.3	
	4	2.65±0.02	
	5	2.41±0.05	
	6	2.52±0.09	
	7	3.3±0.2	
	8	3.34±0.01	
	9	3.11±0.06	
	10	2.48±0.05	
	11	3.9±0.4	
	12	6.8±0.2	
	13	5.1±0.2	
	14	4.63±0.05	
	15	6.8±0.7	

**Fig. 16:**
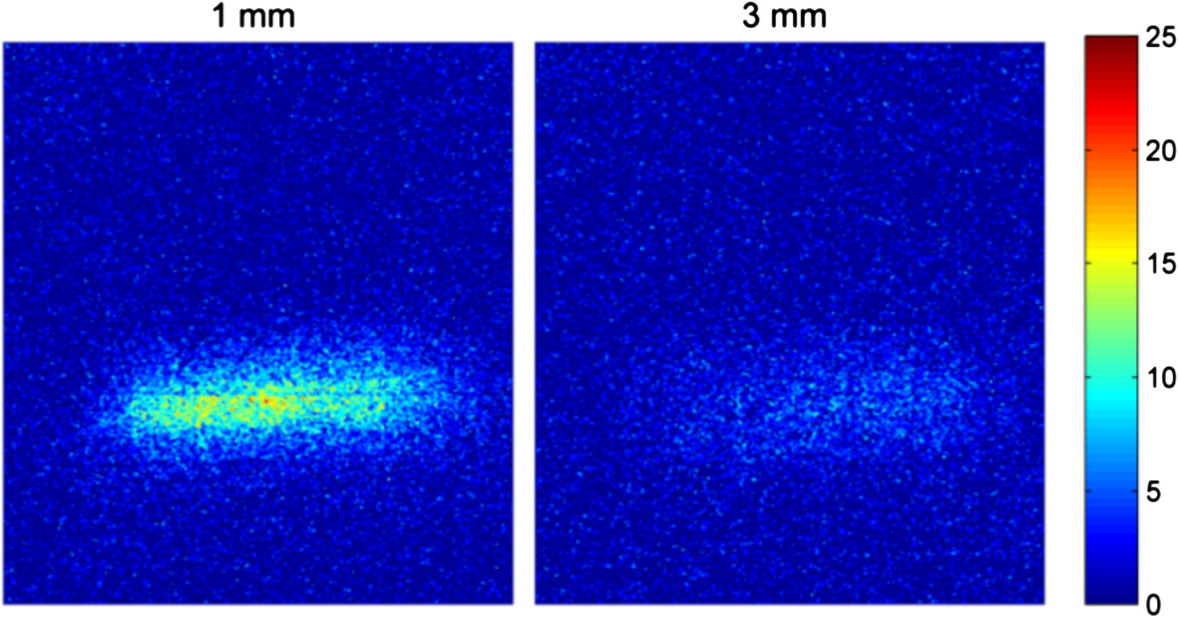
Sample images. Images of the capillary tube filled with ^90^Y placed under slices of chicken breast of 1 mm (*left*) and 3 mm (*right*) thickness, after background subtraction and median filtering. The color scale represents the true source count rate in cps

The images of the capillary tube placed under and 1 and 3 mm of chicken breast are shown in Fig. 16, after the cell-by-cell background subtraction and the median filter application. The images are shown with the same false color scale for the true source count rate (cps) for comparison.

## Discussion

### Advantages of PDPC

As already mentioned, the advantage of silicon photomultipliers with respect to the currently used CCD-based detectors is the single photon counting capability, with potential quantitative capabilities. The additional benefit of the PDPCs over their analog counterpart is the capability to activate a single cell at a time; this reduces the tile active area but allows to monitor the signal and the noise in each cell. In particular, the signal-to-noise ratio can be increased by identifying and turning off the HDC cells in the tile, either at run time during the conventional event-triggered acquisition or in the off-line calculations in DCM mode, as done in this work. This is a capability that is missing so far in analog SiPMs and which can be very useful for low-light-level applications like the detection of Cherenkov radiation. In addition, the PDPCs provide directly the photon count values in terms of number of triggered cells.

### Choice of inhibition threshold *p*

As shown in Fig. 7, inhibition thresholds smaller than 10 % still result in a high dark count rate, while for values greater than 25 % we considered the dark noise reduction (less than 30 % going from *p*=25 *%* to *p*=50 *%*) insufficient to justify the significant loss in terms of active area. Nonetheless, for *p*>25 *%* the differences between the dark count rate curves with temperature become relevant, making the correction algorithm less reliable. The DCR variations with temperature have been studied on a broader range of temperature by Somlai-Schweiger et al. [27], and our results agree with theirs on the limited range of our study (after units matching). The values shown in Fig. 7 for a cell agree also with the ones shown for a pixel in the device manual [28] (Fig. 5.2 of version 0.21), after rescaling our results for the number of cells in a pixel (64×50 cells).

### Correction algorithm

The analysis of the variation of the HDC cells showed that the cells with the highest dark current can be repeatedly identified, since for useful percentages of inhibition (10 *%*≤*p*≤25 *%*), only a small fraction of cells were not re-selected in each dark data set (Table 1). The difference in the distribution of the dark and source count rates was visible even before any correction (Fig. 8), suggesting that the signal could be separated from the background. However, a direct comparison of the two distributions is possible only with a set of measurements with no source at the same exact temperature of the source measurements. This task is difficult to perform and time consuming; therefore, temperature modeling of the dark count rate is needed. For these reasons, we developed an algorithm to select and remove a percentage of HDC cells and to subtract the contribution of dark noise from the source data. We performed a consistency test using separate calibration and test data sets of the dark count rates, thus showing that our baseline restoration works properly (no true source count rate when applied to no source test data, Fig. 11). The application of the HDC cells removal algorithm to the source maps allows us to separate the two populations (Fig. 12). Although the tile active area is reduced by 25 *%*, the estimation of the source count rate is improved by reducing the dark count rate by an order of magnitude (*y* scale in top and bottom plots of Fig. 12). Further improvements could be achieved by lowering the temperature.

### Approximation for DCR dependence on temperature

In the range of temperature of interest (10–13 °C), the dark count rate in the PDPC is known to double approximately every 7.5 °C. In [29], it has been demonstrated that, for a class of analog SiPMs, the dark count rate is described by $DCR \propto T^{1.5}\cdot \mathrm {e}^{-a/T}$, and we expect a similar behavior for the PDPCs. However, on the small range of temperature of our acquisitions, the linear approximation was assumed to be valid. The values of the fit coefficients for the dark count rate as a function of temperature depend on the inhibition percentage *p* (Table 3) since disabling a greater portion of cells corresponds to cutting out the tail of the distribution of count rate of Fig. 8, which is responsible for the increase of the average count rate values. Negative values of count rates at *T*=0 °C are returned by the linear approximation chosen, which is not valid over a broad range of temperatures.

### Effect of chicken breast

Our results with the tissue slices (Fig. 13) show that the signal is stronger for the source alone, indicating that most of the Cherenkov radiation is produced in the source itself and that the tissue mainly plays the role of an attenuator. However, even with almost 1 cm of chicken breast, the count rate is still greater than zero. The comparison of Fig. 13 with the consistency test in Fig. 11, right suggests that this is not due to an incorrect background subtraction. Therefore, we think that the Cherenkov radiation produced in the source might not be completely attenuated by the tissue, and that a part of it could be produced in the chicken breast after high energy photon interactions. It is not trivial to model the attenuation of visible light in an opaque medium like chicken breast to determine the expected attenuation length [30], since Lambert-Beer law can not be applied to our setup, and this is particularly true for Cherenkov radiation, whose production complicates the model. Therefore, quantitative assessment regarding the production and attenuation of Cherenkov radiation in our setup were not done at this stage.

However, we assume that the Cherenkov radiation is mainly produced inside the source container by the different types of particles described, since Cherenkov radiation production outside of the source volume results from a chain of processes (high-energy photons that release photoelectrons or Compton electrons with energy above the Cherenkov threshold) that makes the Cherenkov emission less likely (it should be noted that this secondary radiation has been successfully used, for example for high time resolution Cherenkov TOFPET systems [31], even if in different experimental conditions that favor this chain of processes). As far as the attenuation, the source container attenuates the Cherenkov radiation (Plexiglas transmits approximately 90 % of the visible light), and the container/air interface causes an additional loss of light due to reflection and refraction. The addition of a layer of tissue with no optical coupling results in two additional interfaces that refract and reflect the light produced in the source container. A maximum penetration depth of 1.9 cm for visible light in chicken breast was measured in [32]. From this measured value, we can expect roughly a 42 % reduction of the light intensity over 8 mm, which is in good agreement with our measured 40 % reduction.

### Effect of other materials

The results of the test with the different shields (Fig. 14) suggest that the detected signal is actually the Cherenkov radiation, and that the only effective shield is the black Tedlar sheet. Switching the two materials showed no visible effect. The same considerations done for the setup with the tissue apply to the case of the Plexiglas shield, but in this case, we expect a higher production and transmission in the external material. Based on the results of the model in [14], that compare the relative intensities of the different components of the signal, we expect that also the signal produced in the source volume mostly comes from the primary *β*^+^, and only a small part is due to secondary particles. A small fraction of the measured count rate (both with the tissue and the shields) could be due to direct detection of high energy photons, this and the additional Cherenkov emission could be reduced by choosing a pure *β*^−^ emitters, like ^90^Y.

### Count rate linearity with activity and imaging capabilities

Finally, the PDPC detector in DCM mode demonstrated a linear response of the measured count rate with the source activity. It also showed imaging capabilities with a spatial resolution (limited by cell size) that can not be achieved in the conventional event-triggered mode (like with analog SiPMs), where the lower limit to the spatial resolution is the pixel pitch. However, the main limitation to the spatial resolution still comes from the light scattering and absorption in the tissue, like in CCD-based acquisitions, and can only be overcome with a proper inverse model for the light transport.

### Corrections for quantitative measurements

In evaluating which effects should be estimated and compensated for quantitative measurements, we qualitatively observed that dead time is dominated by a fixed read-out time. Nonetheless, an estimation of dead time variability should be made to determine if compensation methods are needed. We also noted that the tile temperature increases rapidly after being powered up and stabilizes after approximately 3 min, consistent with the results of Somlai-Schweiger et al. [27]. This implies a variation in the signal to noise ratio as a function of the time of cell firing during the acquisition in DCM mode. Any confounding effect might be mitigated by grouping together the cells that are activated in parallel (rather than dies) and employing a predetermined correction based on the time from power-up. The extent of the errors introduced by this effect are not yet known, although we believe they would not impact any of the results presented here. Another potential source of error that we did not evaluate is the use of one DCM to select the HDC cells in the other maps, as this neglects the effect of temperature differences between the maps. Finally, the variation of the effect of the inhibition level between dies was not presented here, but is available in the additional material. The comparison of the dark count rate measured without and with the lens (Table 5) suggests that also the light tightness of the chamber should be thoroughly tested to provide reliable quantitative measurements of photon count values.

A limitation of the DCM acquisition mode is the fact that the cells are read sequentially, increasing the acquisition time and reducing the available active area. In fact, during the DCM measurement, Cherenkov photons are lost when they reach a sensor cell outside the single cell activation time. However, the total acquisition time chosen in this study was the same as typical CCD-based previous studies (on the order of few minutes). The signal-to-noise ratio obtained in the same time is probably different due to the different working principle of the CCD (an integrating device) with respect to the SiPM (a single photon counting device), but the spatial resolution achievable when reading one cell at a time is comparable to that of CCD-based optical systems (considering the pixel binning that is usually applied, see for example [6, 11, 13]), and the single photon counting capability suggests better potential quantitative capabilities. A system able to read all the cells of a single photon counting device at the same time would be optimal.

Due to the complexity of this setup, it is very useful to understand the different contributions to the global signal. On the other hand, since the Cherenkov radiation is produced almost entirely in the source volume, it is difficult to perform quantitative intensity measurements as a function of the volume of Cherenkov radiator. To this aim, a simpler setup with a more homogeneous material could be employed, for example diluting the radioactive source in a volume of water and varying the volume and/or the concentration of radioisotope. A pure *β*^−^ emitter would reduce the number of possible processes resulting in Cherenkov emission, thus simplifying the modeling.

In an application like preclinical imaging, the small animal would be both the source and the attenuator. The number of detected photons, together with their spatial distribution on the PDPC tile and the detector photon detection efficiency should be able to provide the number of photons impinging on the photodetector. From there, the optical transport of the Cherenkov radiation should be modeled to obtain the number of Cherenkov photons produced. As a final consideration, we think that the results of this study could be extended to other low-light levels applications, suggesting that the PDPC could be used also to detect faint bioluminescence signals or other low intensity sources. To this aim, a dedicated study with a calibrated low-intensity source would be useful.

## Conclusions

We showed that a PDPC detector, at a temperature of (10–13) °C and reading one cell at a time in DCM mode, is able to detect and to measure the Cherenkov radiation produced in the decay of a *β* emitter. The signal can be more readily separated from the background by switching off a certain fraction of cells with the highest dark current and taking the temperature of the measurement into account to subtract the expected dark count rate from the measured count rate. The results of this operation could be improved performing the acquisition at lower temperatures. The number of counted photons monotonically decreases with the thickness of the attenuator placed between the source and the detector and follows linearly the source activity, suggesting that the PDPCs have potential quantitative capabilities. Measurements with different shields (black paper for visible radiation and Plexiglas for *β* particles) showed that most of the detected signal is due to the Cherenkov radiation. Finally, the PDPC detector in DCM mode showed imaging capabilities that make this kind of photosensor a potential alternative to CCD devices. The sequential reading of the cells and the temperature control are key issues that would need to be addressed. The results of this study could be easily extended to other low-light levels applications such as bioluminescence.

A next step could be to assess if the developed algorithm for the data collection and processing can be quantitative without any cross-calibration. This could be done by determining the most appropriate percentage of cells to inhibit to increase the signal-to-noise ratio and by trying to estimate the loss of photons due to factors like the sensor dead time and the percentage of inhibition chosen, and by implementing dedicated corrections for each factor. Future work could also focus on the fine spatial sampling of the distribution of the light impinging on the detector when operated in DCM mode.

## Additional files

Additional file 1
**Comparison of the count rate with and without source.** Distribution of the count rate measured by the cells of each die at the same temperature for an acquisition with no source (dotted line, *T*=12.3±0.2 °C) and for an acquisition with the source (solid line, *T*=12.2±0.4 °C). All the cells were considered (no HDC cells inhibition). The plot was truncated to show details al lower count rates. Labels and legend follow Fig. 8, and plot positions refer to die positions in the die (Fig. 2-center). (PNG 54 kb)

Additional file 2
**Comparison of measurements at same temperature.** Distribution of the differences in the count rates measured in two independent acquisitions performed at the same temperature (12.5±0.4 °C), for all dies. Labels and legend follow Fig. 9, and plot positions refer to die positions in the die (Fig. 2-center). (PNG 44 kb)

Additional file 3
**Comparison of the count rate with different activation time.** Labels and legend follow Fig. 10, and plot positions refer to die positions in the die (Fig. 2-center). (PNG 49 kb)

Additional file 4
**Test of correction algorithm, left.** Application of the correction algorithm to the test set without the source, for all dies. Error bars are taken from Table 2. Labels and legend follow Fig. 11-left, and plot positions refer to die positions in the die (Fig. 2-center). (PNG 54 kb)

Additional file 5
**Test of correction algorithm, right.** Application of the correction algorithm to the test set without the source, for all dies. Error bars are taken from Table 2. Labels and legend follow Fig. 11-right, and plot positions refer to die positions in the die (Fig. 2-center). (PNG 31 kb)

Additional file 6
**Effect of the HDC cells removal algorithm,** ***p*** **= 0 %.** Labels and legend follow Fig. 12, and plot positions refer to die positions in the die (Fig. 2-center). (PNG 50 kb)

Additional file 7
**Effect of the HDC cells removal algorithm,** ***p*** **= 10 %.** Labels and legend follow Fig. 12, and plot positions refer to die positions in the die (Fig. 2-center). (PNG 53 kb)

Additional file 8
**Effect of the HDC cells removal algorithm,** ***p*** **= 25 %.** Labels and legend follow Fig. 12, and plot positions refer to die positions in the die (Fig. 2-center). (PNG 60 kb)

Additional file 9
**Effect of attenuator,** ***p*** **= 10 %.** Effect of the attenuation of the chicken breast placed between source and detector, with the setup shown in Fig. 3 in all dies. Labels and legend follow Fig. 13, and plot positions refer to die positions in the die (Fig. 2-center). (PNG 41 kb)

Additional file 10
**Effect of attenuator,** ***p*** **= 25 %.** Effect of the attenuation of the chicken breast placed between source and detector, with the setup shown in Fig. 3 in all dies. Labels and legend follow Fig. 13, and plot positions refer to die positions in the die (Fig. 2-center). (PNG 40 kb)

Additional file 11
**Effect of materials.** Labels and legend follow Fig.14, and plot positions refer to die positions in the die (Fig.2-center). (PNG 31 kb)

Additional file 12
**Linearity of count rate with activity.** TSCR measured in one central die as a function of the source activity at the time of the measurements (circle). Linear fit of Eq. 4 (solid line). Labels and legend follow Fig. 15, and plot positions refer to die positions in the die (Fig. 2-center). (PNG 42 kb)
