# Supplementary figures and images for: Cherenkov luminescence measurements with digital silicon photomultipliers: a feasibility study
Source: EJNMMI Phys. 2015 Nov 16;2:32. doi: 10.1186/s40658-015-0134-z (PMC4646894; doi:10.1186/s40658-015-0134-z)

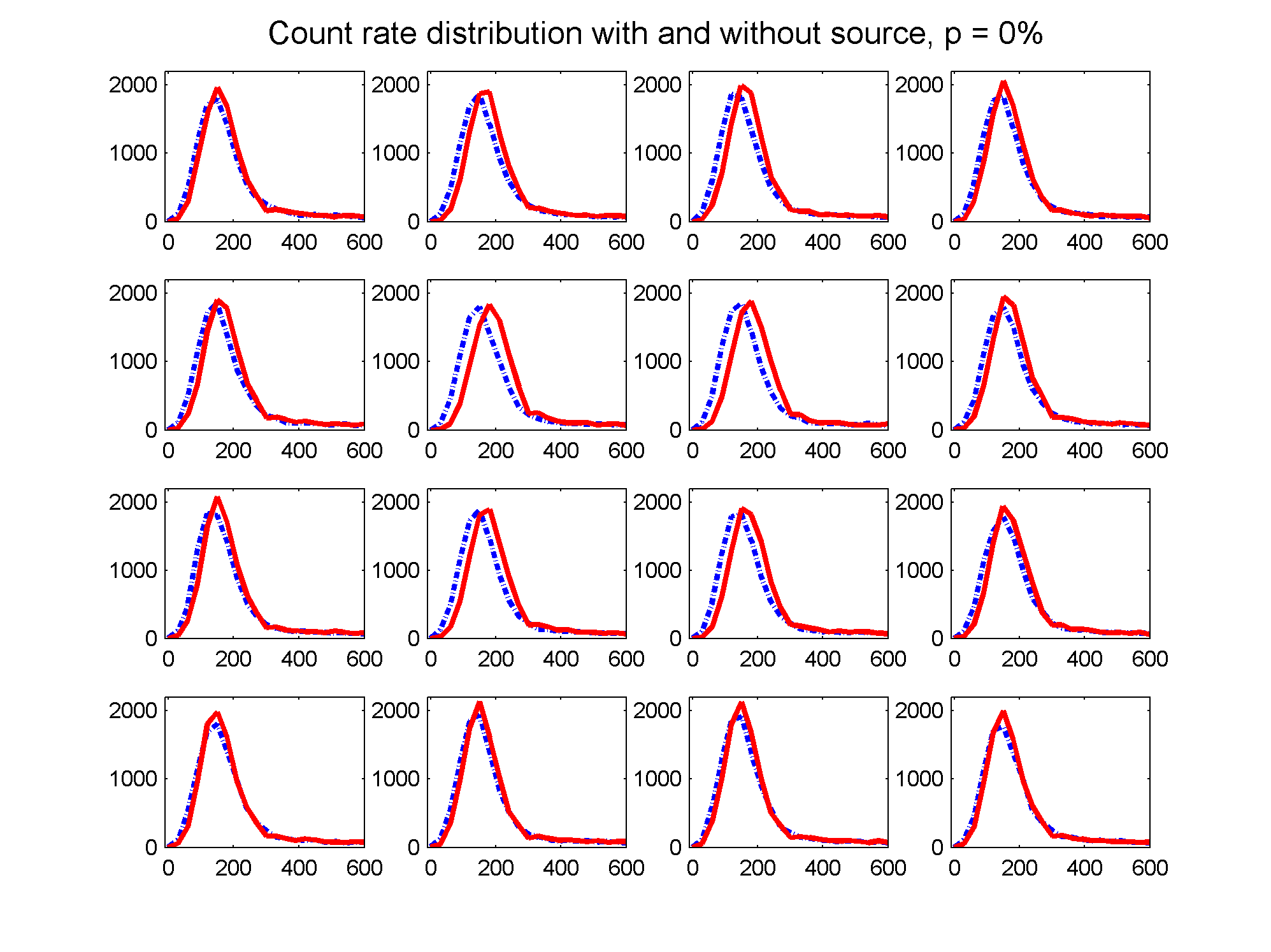

Supplement: Additional file 1 — Comparison of the count rate with and without source. Distribution of the count rate measured by the cells of each die at the same temperature for an acquisition with no source (dotted line, T=12.3±0.2 °C) and for an acquisition with the source (solid line, T=12.2±0.4 °C). All the cells were considered (no HDC cells inhibition). The plot was truncated to show details al lower count rates. Labels and legend follow Fig. 8, and plot positions refer to die positions in the die (Fig. 2-center). (PNG 54 kb) [file 40658_2015_134_MOESM1_ESM.png]

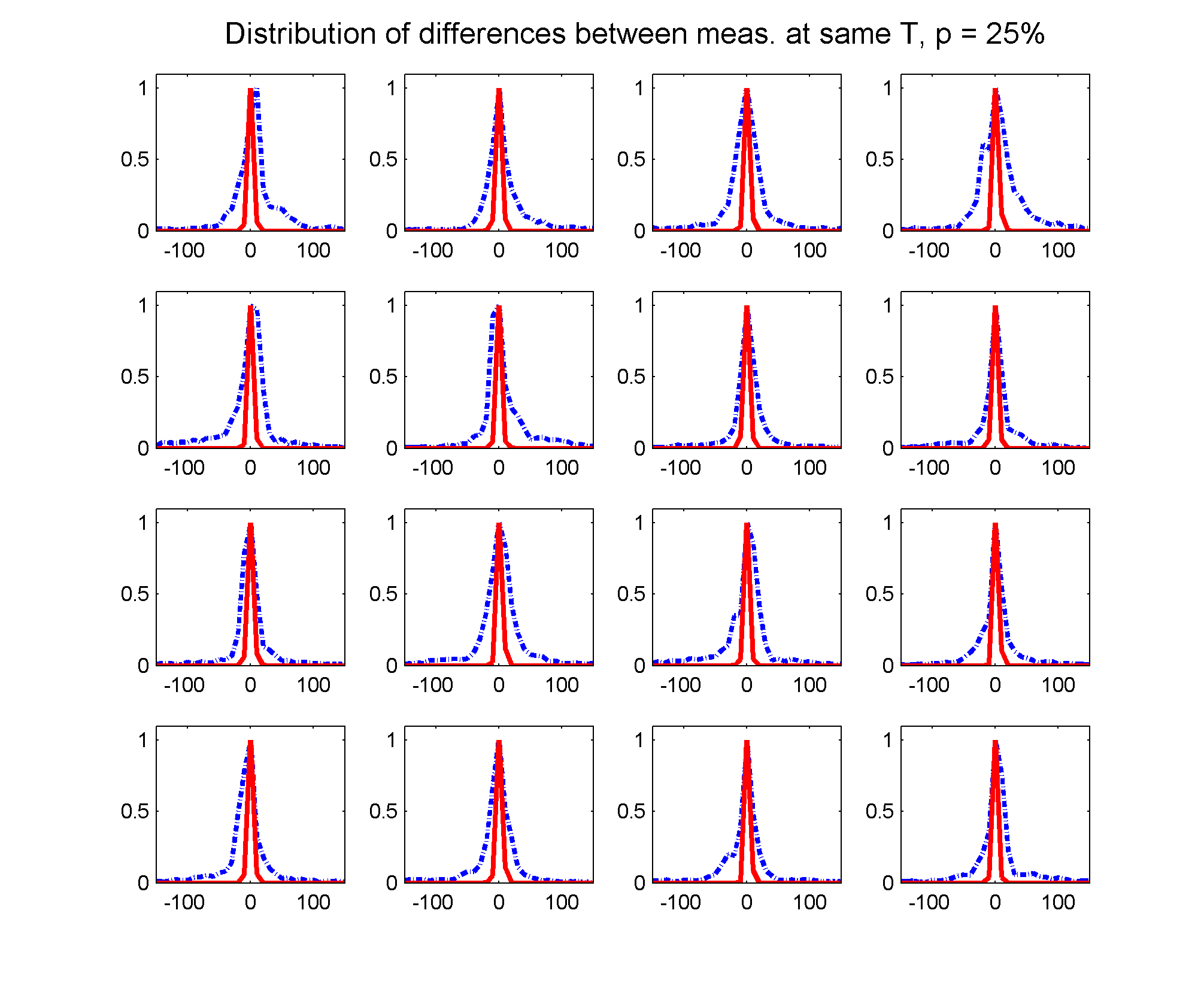

Supplement: Additional file 2 — Comparison of measurements at same temperature. Distribution of the differences in the count rates measured in two independent acquisitions performed at the same temperature (12.5±0.4 °C), for all dies. Labels and legend follow Fig. 9, and plot positions refer to die positions in the die (Fig. 2-center). (PNG 44 kb) [file 40658_2015_134_MOESM2_ESM.png]

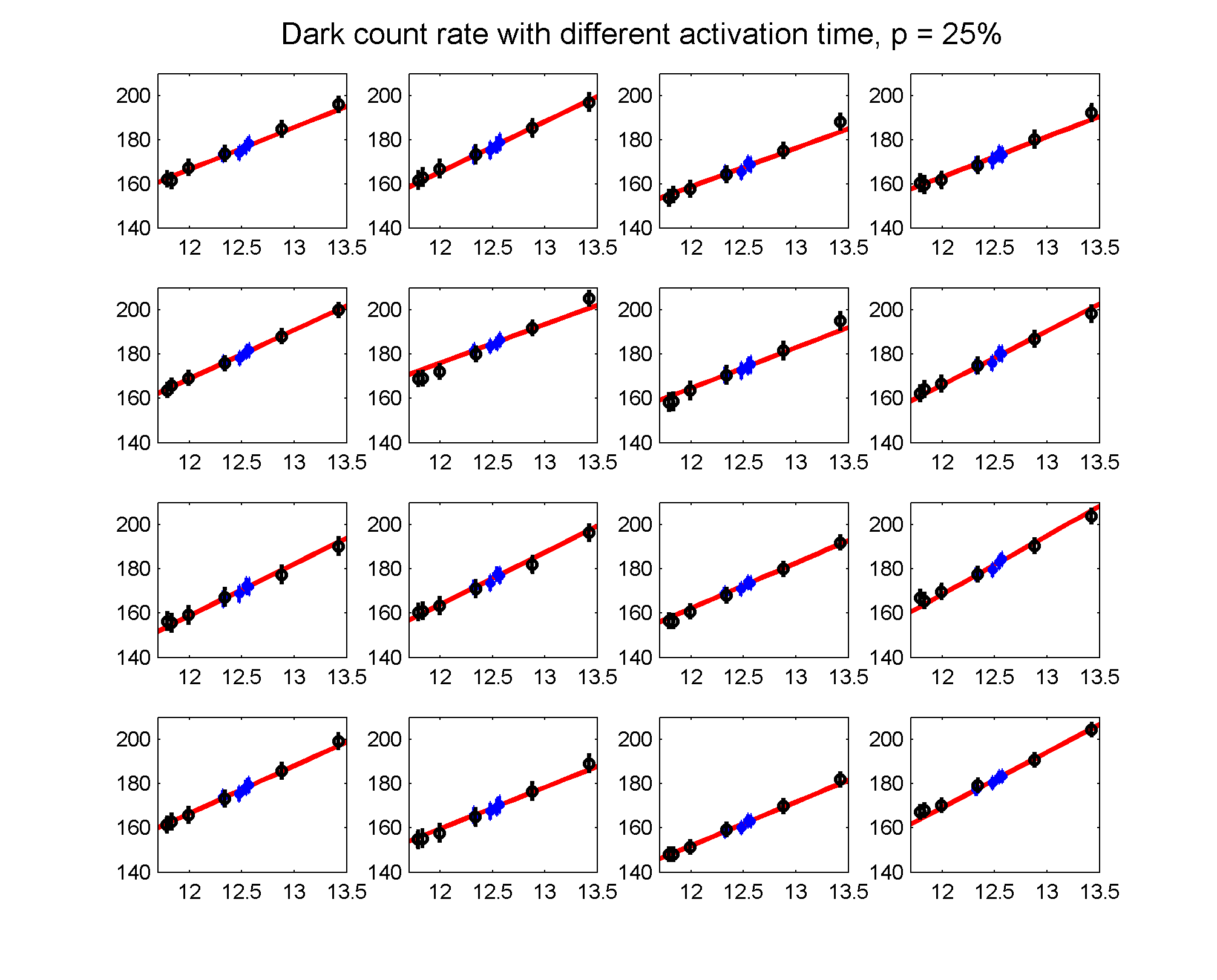

Supplement: Additional file 3 — Comparison of the count rate with different activation time. Labels and legend follow Fig. 10, and plot positions refer to die positions in the die (Fig. 2-center). (PNG 49 kb) [file 40658_2015_134_MOESM3_ESM.png]

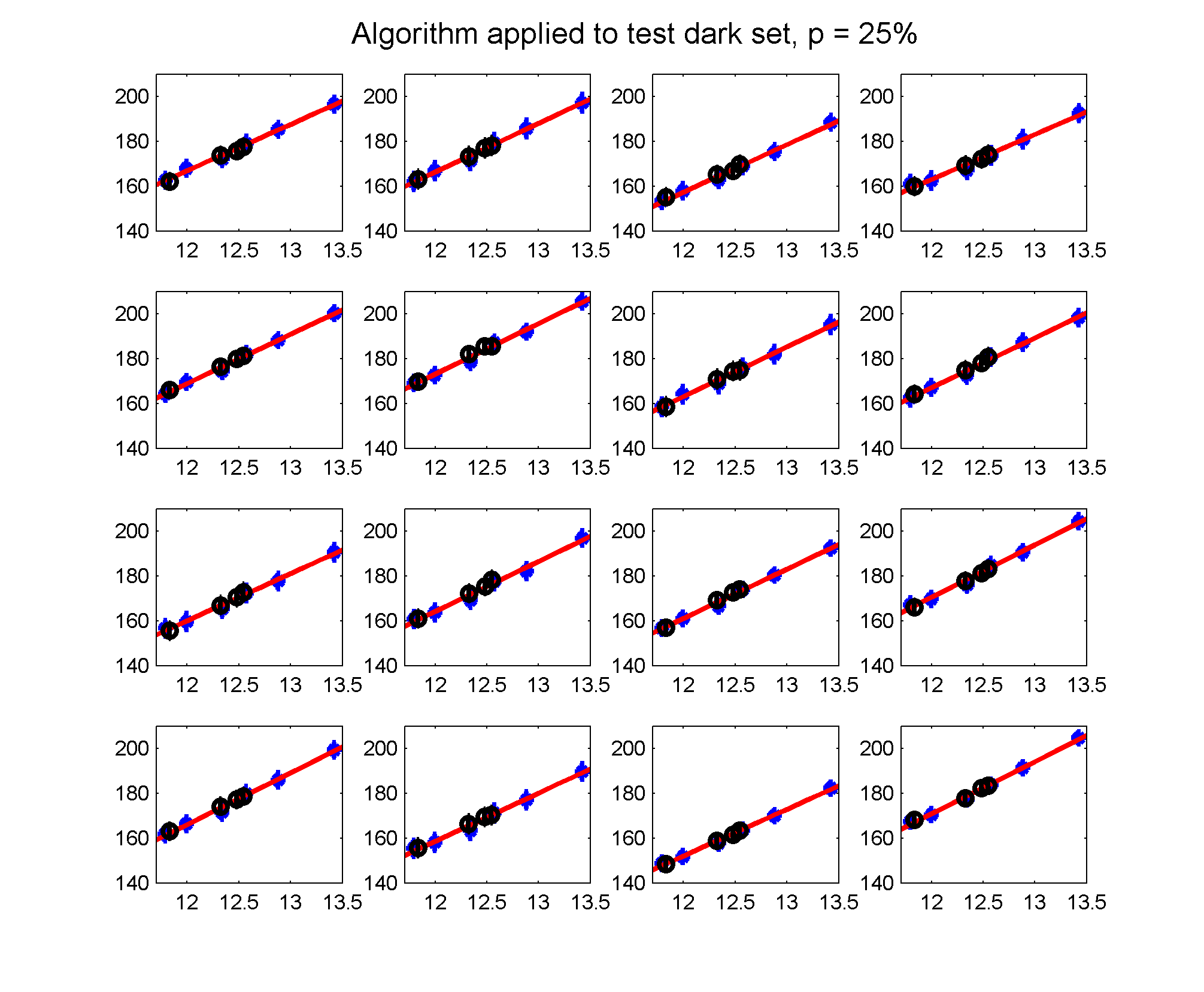

Supplement: Additional file 4 — Test of correction algorithm, left. Application of the correction algorithm to the test set without the source, for all dies. Error bars are taken from Table 2. Labels and legend follow Fig. 11-left, and plot positions refer to die positions in the die (Fig. 2-center). (PNG 54 kb) [file 40658_2015_134_MOESM4_ESM.png]

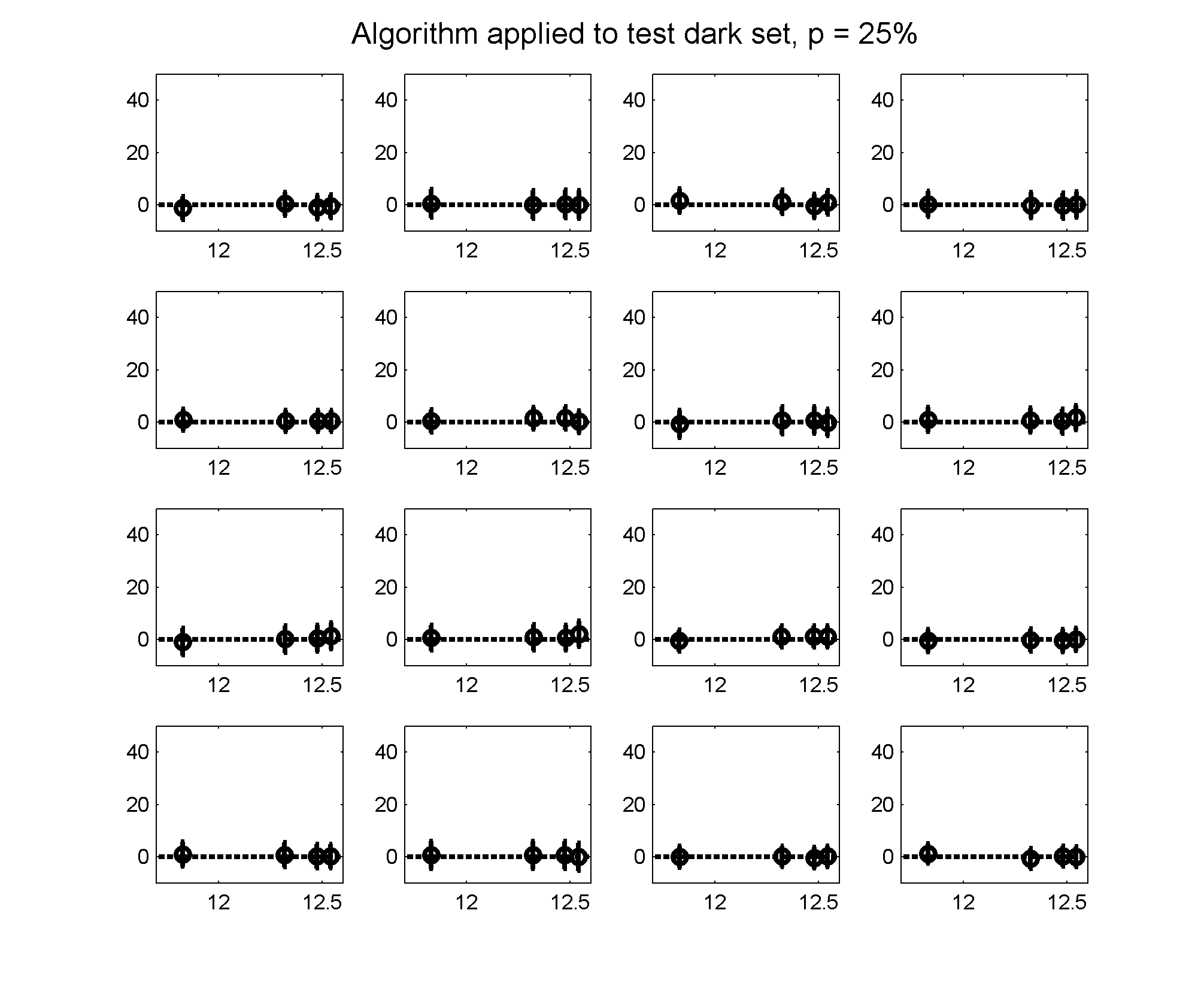

Supplement: Additional file 5 — Test of correction algorithm, right. Application of the correction algorithm to the test set without the source, for all dies. Error bars are taken from Table 2. Labels and legend follow Fig. 11-right, and plot positions refer to die positions in the die (Fig. 2-center). (PNG 31 kb) [file 40658_2015_134_MOESM5_ESM.png]

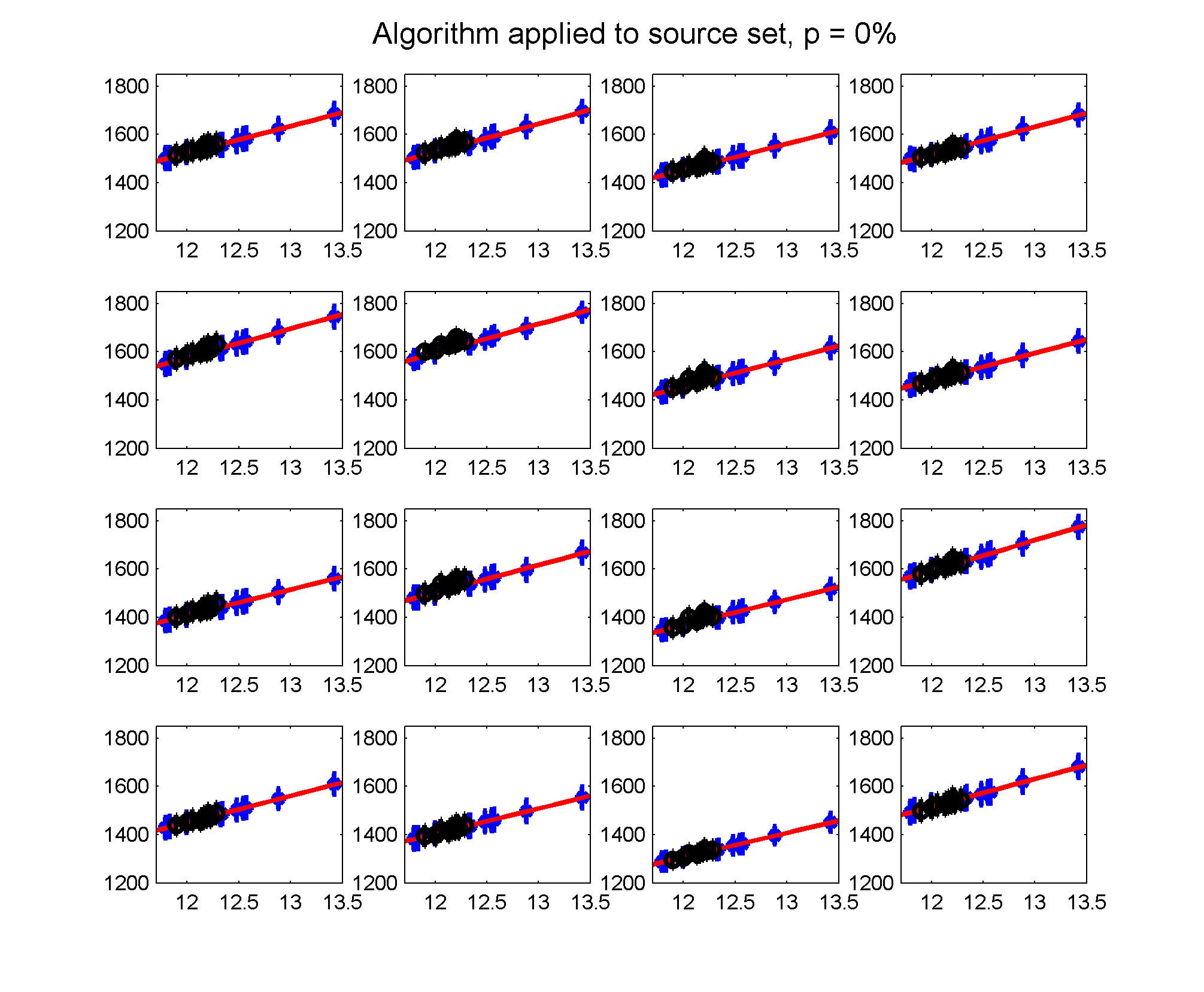

Supplement: Additional file 6 — Effect of the HDC cells removal algorithm, p = 0 %. Labels and legend follow Fig. 12, and plot positions refer to die positions in the die (Fig. 2-center). (PNG 50 kb) [file 40658_2015_134_MOESM6_ESM.png]

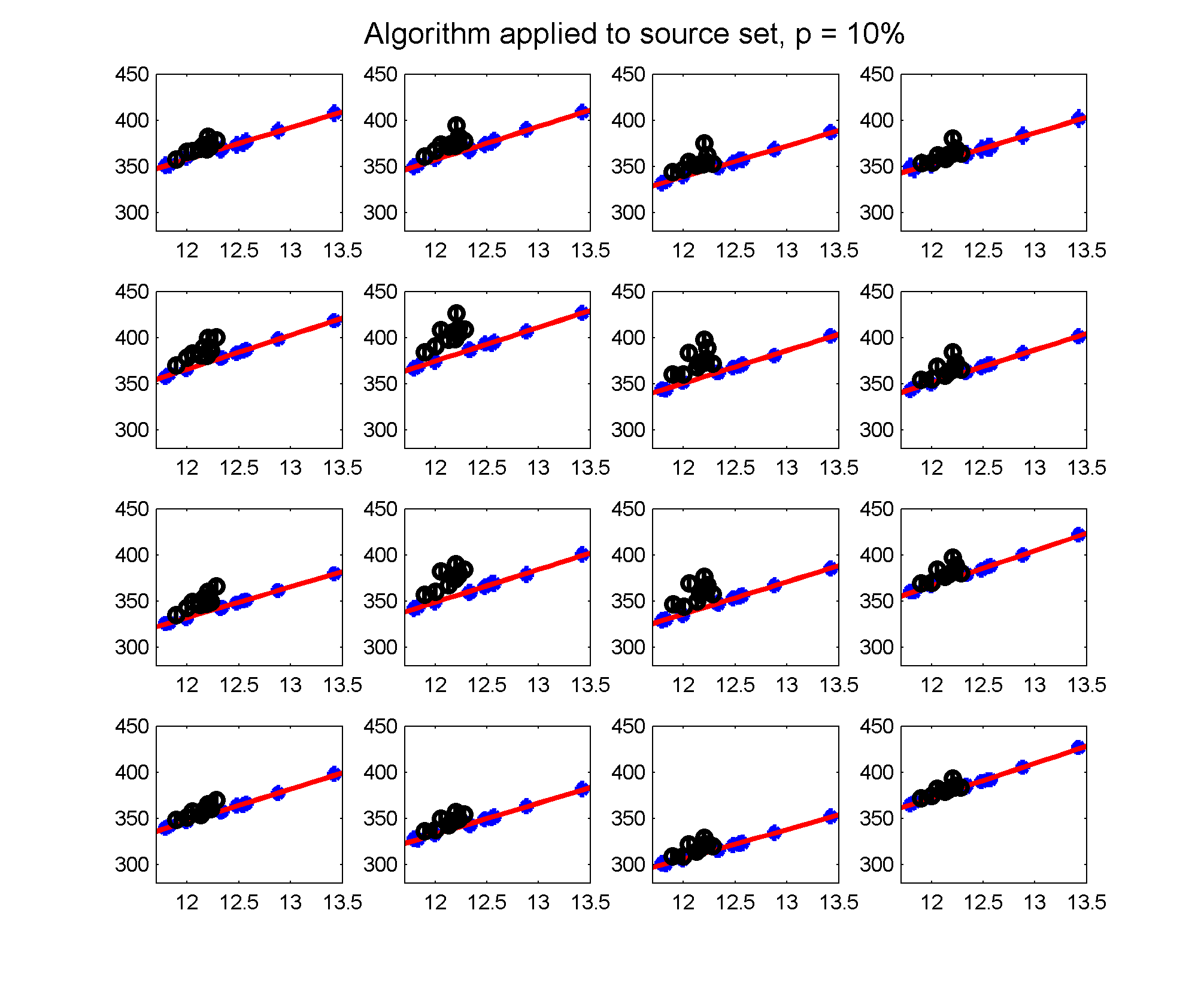

Supplement: Additional file 7 — Effect of the HDC cells removal algorithm, p = 10 %. Labels and legend follow Fig. 12, and plot positions refer to die positions in the die (Fig. 2-center). (PNG 53 kb) [file 40658_2015_134_MOESM7_ESM.png]

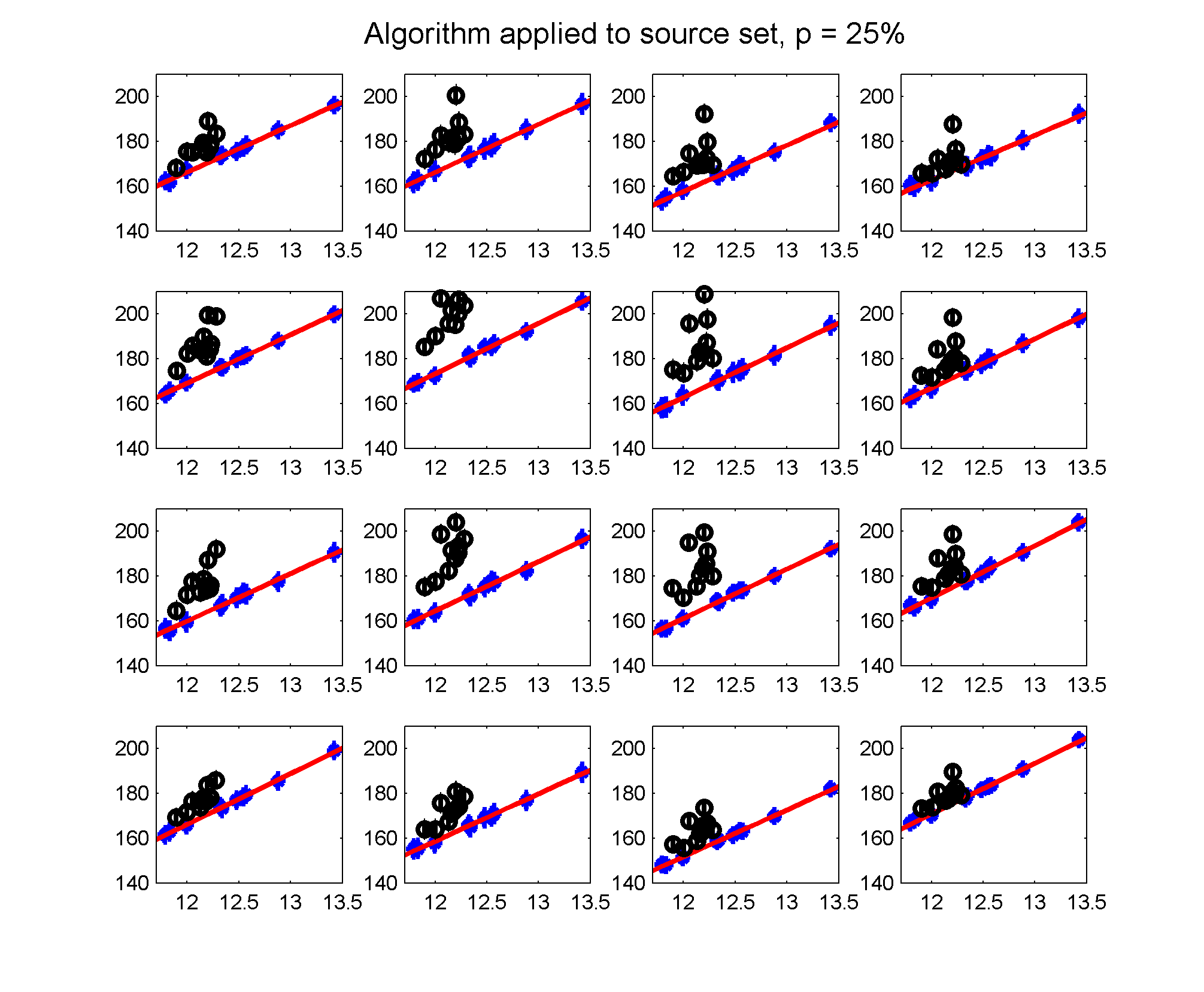

Supplement: Additional file 8 — Effect of the HDC cells removal algorithm, p = 25 %. Labels and legend follow Fig. 12, and plot positions refer to die positions in the die (Fig. 2-center). (PNG 60 kb) [file 40658_2015_134_MOESM8_ESM.png]

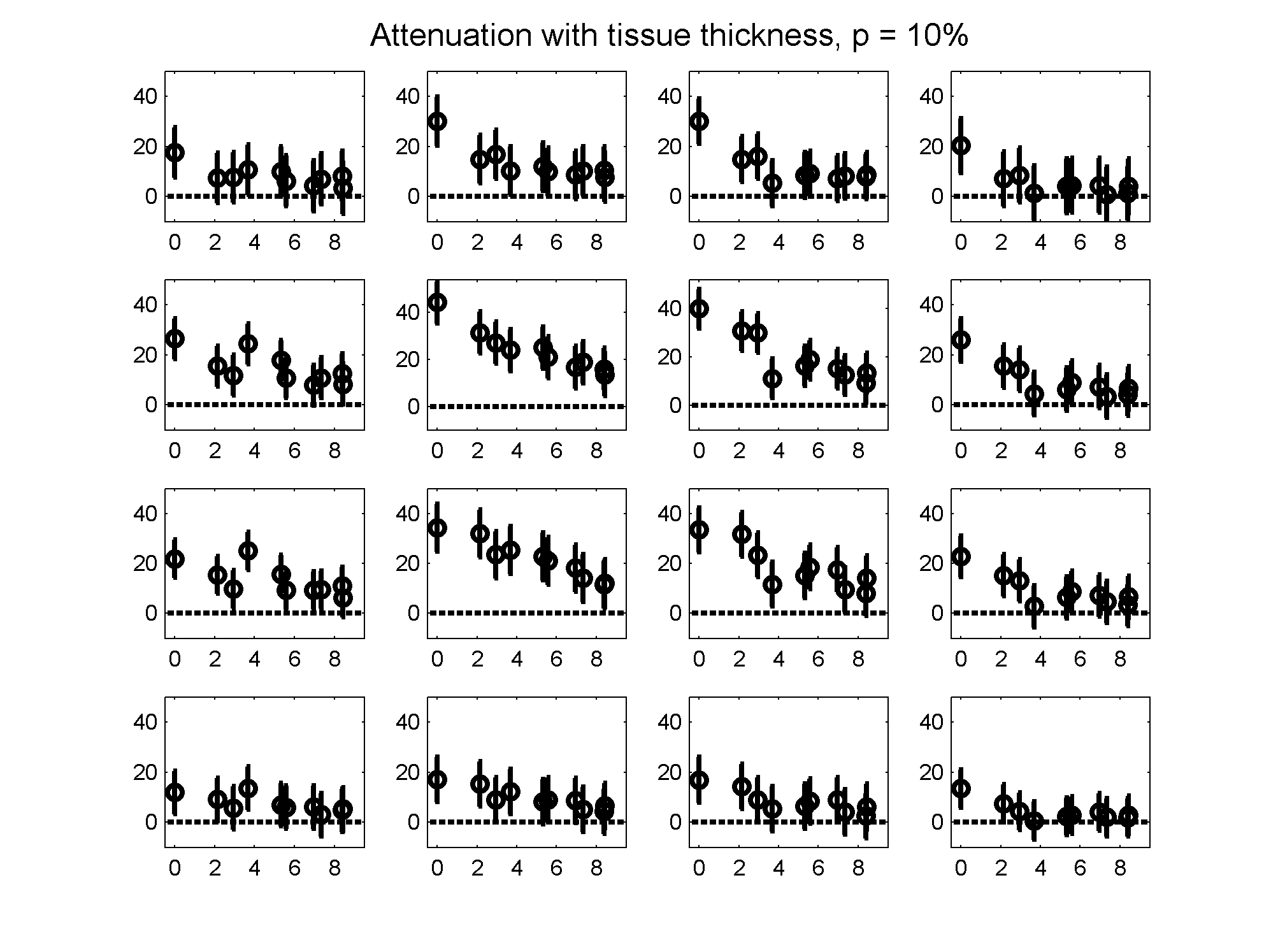

Supplement: Additional file 9 — Effect of attenuator, p = 10 %. Effect of the attenuation of the chicken breast placed between source and detector, with the setup shown in Fig. 3 in all dies. Labels and legend follow Fig. 13, and plot positions refer to die positions in the die (Fig. 2-center). (PNG 41 kb) [file 40658_2015_134_MOESM9_ESM.png]

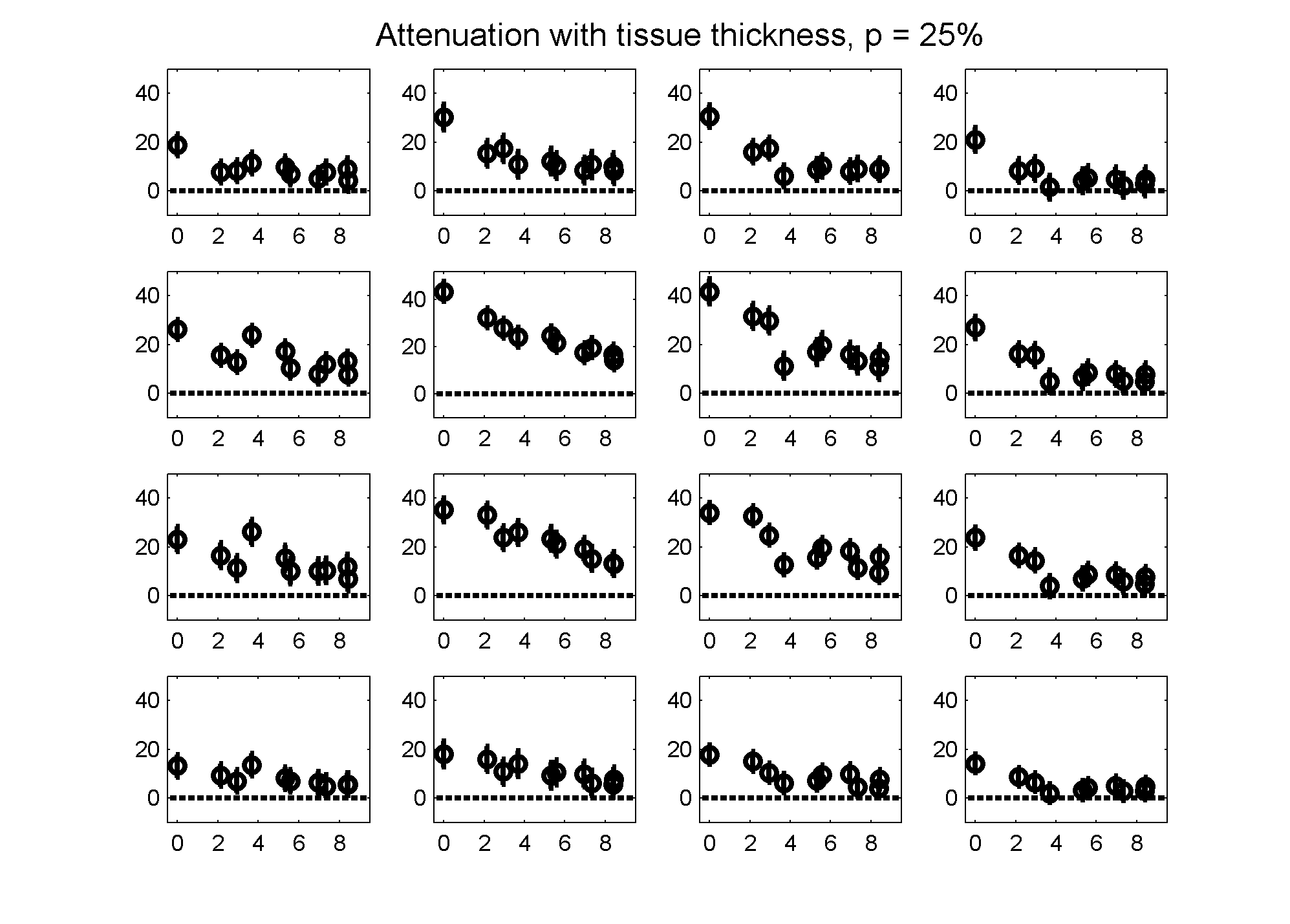

Supplement: Additional file 10 — Effect of attenuator, p = 25 %. Effect of the attenuation of the chicken breast placed between source and detector, with the setup shown in Fig. 3 in all dies. Labels and legend follow Fig. 13, and plot positions refer to die positions in the die (Fig. 2-center). (PNG 40 kb) [file 40658_2015_134_MOESM10_ESM.png]

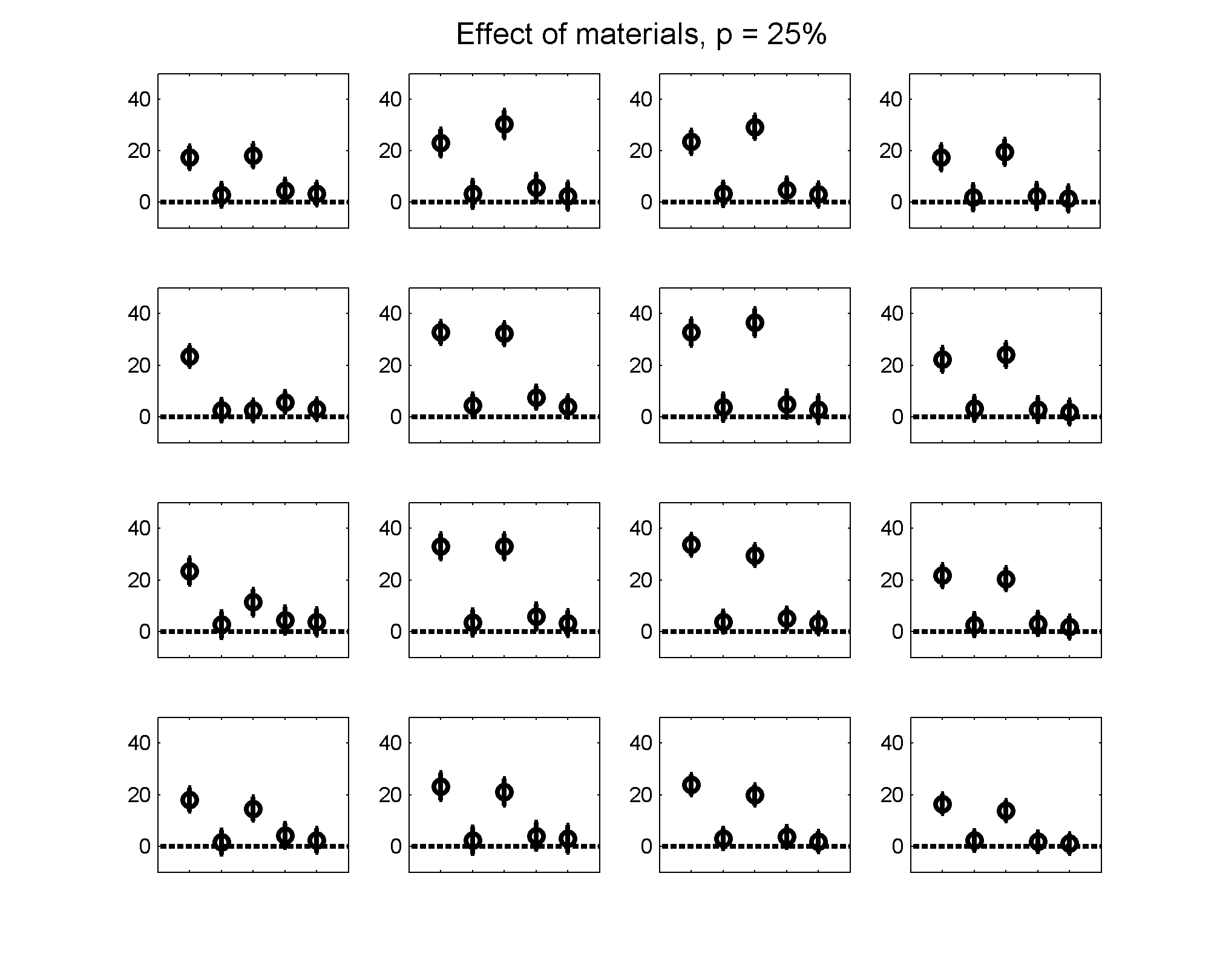

Supplement: Additional file 11 — Effect of materials. Labels and legend follow Fig.14, and plot positions refer to die positions in the die (Fig.2-center). (PNG 31 kb) [file 40658_2015_134_MOESM11_ESM.png]

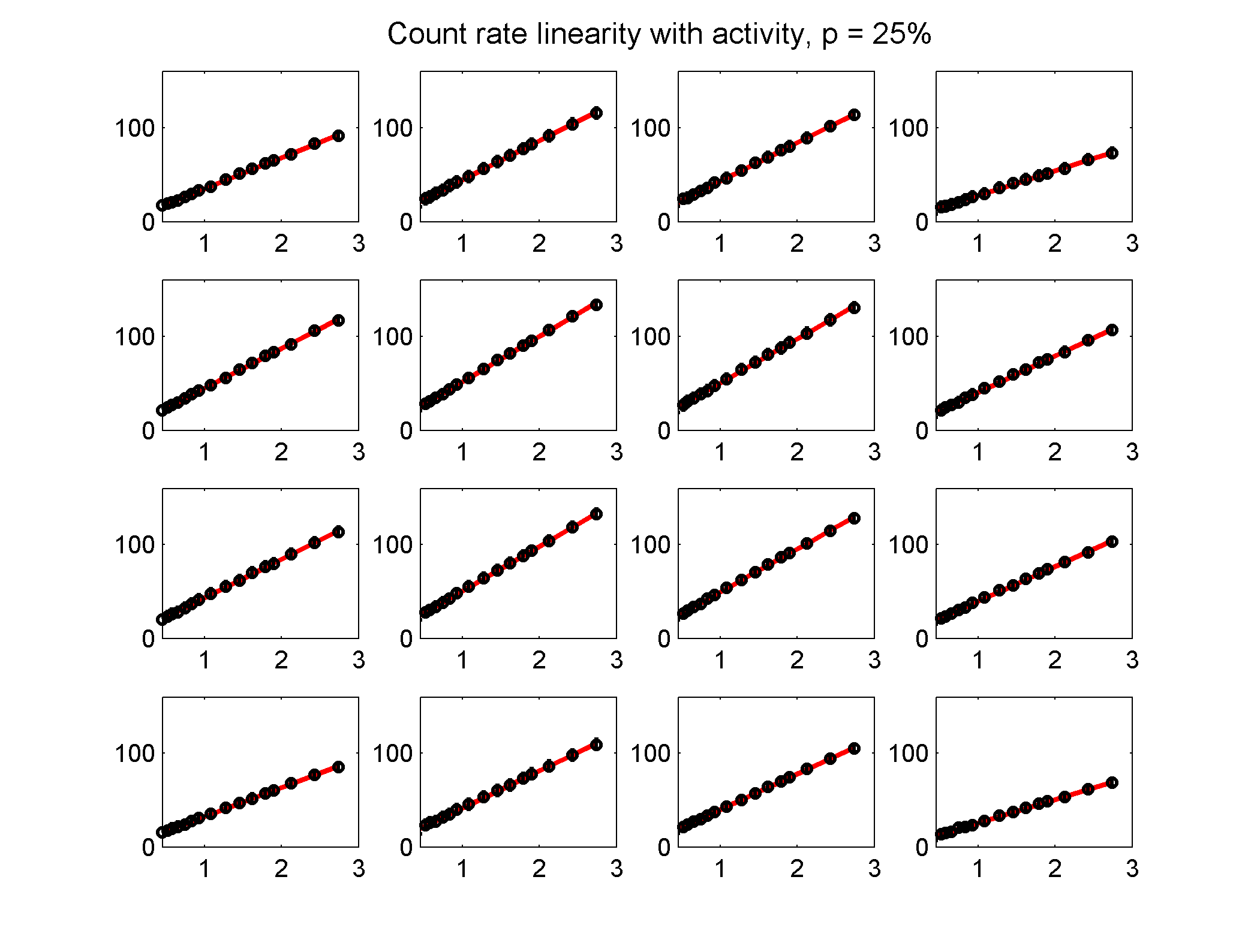

Supplement: Additional file 12 — Linearity of count rate with activity. TSCR measured in one central die as a function of the source activity at the time of the measurements (circle). Linear fit of Eq. 4 (solid line). Labels and legend follow Fig. 15, and plot positions refer to die positions in the die (Fig. 2-center). (PNG 42 kb) [file 40658_2015_134_MOESM12_ESM.png]
